# On the use of whole-genome sequence data for across-breed genomic prediction and fine-scale mapping of QTL

**DOI:** 10.1186/s12711-021-00607-4

**Published:** 2021-02-26

**Authors:** Theo Meuwissen, Irene van den Berg, Mike Goddard

**Affiliations:** 1 Norwegian University of Life Sciences, Box 5003, 1432 Ås, Norway; 2 Agriculture Victoria, Bundoora, Australia; 3 Faculty of Veterinary and Agricultural Sciences, University of Melbourne, Parkville, Australia

## Abstract

**Background:**

Whole-genome sequence (WGS) data are increasingly available on large numbers of individuals in animal and plant breeding and in human genetics through second-generation resequencing technologies, 1000 genomes projects, and large-scale genotype imputation from lower marker densities. Here, we present a computationally fast implementation of a variable selection genomic prediction method, that could handle WGS data on more than 35,000 individuals, test its accuracy for across-breed predictions and assess its quantitative trait locus (QTL) mapping precision.

**Methods:**

The Monte Carlo Markov chain (MCMC) variable selection model (Bayes GC) fits simultaneously a genomic best linear unbiased prediction (GBLUP) term, i.e. a polygenic effect whose correlations are described by a genomic relationship matrix (**G**), and a Bayes C term, i.e. a set of single nucleotide polymorphisms (SNPs) with large effects selected by the model. Computational speed is improved by a Metropolis–Hastings sampling that directs computations to the SNPs, which are, a priori, most likely to be included into the model. Speed is also improved by running many relatively short MCMC chains. Memory requirements are reduced by storing the genotype matrix in binary form. The model was tested on a WGS dataset containing Holstein, Jersey and Australian Red cattle. The data contained 4,809,520 genotypes on 35,549 individuals together with their milk, fat and protein yields, and fat and protein percentage traits.

**Results:**

The prediction accuracies of the Jersey individuals improved by 1.5% when using across-breed GBLUP compared to within-breed predictions. Using WGS instead of 600 k SNP-chip data yielded on average a 3% accuracy improvement for Australian Red cows. QTL were fine-mapped by locating the SNP with the highest posterior probability of being included in the model. Various QTL known from the literature were rediscovered, and a new SNP affecting milk production was discovered on chromosome 20 at 34.501126 Mb. Due to the high mapping precision, it was clear that many of the discovered QTL were the same across the five dairy traits.

**Conclusions:**

Across-breed Bayes GC genomic prediction improved prediction accuracies compared to GBLUP. The combination of across-breed WGS data and Bayesian genomic prediction proved remarkably effective for the fine-mapping of QTL.

## Background

In animals, plants and humans, data on phenotypes and genome-wide genotypes are used for both genomic prediction and for mapping and identification of the causal variants that affect the phenotype. Whole-genome sequence (WGS) data are increasingly available on large numbers of individuals in animal and plant breeding, and in humans. This is due to cost-effective second-generation resequencing technologies, in combination with 1000 genomes projects (e.g. for humans [1]; plants [2]; and livestock [3]). The 1000 genomes projects in combination with modern genotype imputation software (e.g. [4, 5]) mean that single nucleotide polymorphism (SNP) chip data can be imputed to full sequence genotypes for large numbers of individuals.

In genomic prediction, genotypes and phenotypes on individuals in a training population are used to predict the breeding value of individuals in the target population that have genotypes but may not have phenotypes. The accuracy of prediction depends on the size of the training population and the extent of linkage disequilibrium (LD). Populations with extensive LD (e.g. many livestock breeds) require a smaller training population than populations with less LD (e.g. humans; [6]). To maximize the size of the training population, one might consider combining data across breeds of livestock or across human populations. Across-population predictions are especially valuable for small populations, and also when the number of phenotypes per population is small due to recording difficulties. However, accuracy of prediction declines if the target population is not closely related to the training population because the LD between markers (e.g. single nucleotide polymorphisms or SNPs) and causal variants differs between populations. Therefore, a method of genomic prediction that maintains higher accuracy when the training and target populations are not closely related is desirable. Part of such a method would exploit high-density marker or whole-genome sequence (WGS) data because then markers that are close to the causal variants, or the causal variants themselves, are included in the data [7]. However, to make effective use of such high-density markers, a method of variable selection is needed so that the causal variants or markers in high LD with them dominate the prediction.

Genome-wide association data is also used to map and identify these causal variants. While mapping causal variants for complex traits to a chromosomal region is common, identification of the causal variants is less common because the causal variant is likely to be in high LD with many other variants. Thus, to identify them, first, the causal variants must be included in the data and second, statistical methods to identify them are required. Fine-scale mapping often considers genome sequence data within a small chromosomal region, but it would be advantageous to do this across the whole genome. Within a population, long-range LD causes SNPs that are located far from a causal variant to be associated with it, which implies the identification of a broad quantitative trait locus (QTL) region. Genomic selection models with variable selection fit all SNPs simultaneously and thus, they position more precisely and possibly identify the causal variants, especially when data from several populations are combined.

A problem with the use of Bayesian variable selection methods is that they are computationally very intensive because they typically involve Monte Carlo Markov chain (MCMC) sampling. Fast iterative methods for Bayesian genomic prediction have been developed, but they are generally not quite as accurate as their MCMC counterparts [8]. Since the improvements from using WGS data may be small, we cannot afford to lose any accuracy. However, to estimate the effects of millions of SNPs, we need very large numbers of individuals and thus very large datasets, which makes the computational costs of MCMC sampling excessively high. Several methods for improving computational speed of MCMC sampling have been published in the past (e.g. [9, 10]). Here, we present a relatively fast MCMC implementation of a Bayesian variable selection method that can handle WGS data on large numbers of animals. We incorporate several methods to make the MCMC analysis of WGS data computationally more efficient, including Metropolis–Hastings (MH) sampling to direct computational efforts to the most important SNPs, bitwise storage of genotypes in the main memory of the computer, and simultaneous evaluations of several (relatively short) MCMC chains using multiple threads.

In this paper, we present a method for fine-scale mapping and genomic prediction across breeds of cattle using Bayesian variable selection and WGS data on large numbers of individuals. The developed method is called Bayes GC and the model was applied to the three dairy breeds: Holstein (H), Jersey (J), and Australian Red (AR). We will use Bayes GC to map some of the causal variants, to demonstrate a new method for calculating confidence intervals for causal variants, and to compare the accuracy of WGS-based genomic predictions to those obtained using dense 600 k SNP chip data and using the genomic best linear unbiased prediction (GBLUP) method.

## Methods

### Data

The dataset used for this analysis is a subset of the data that are described in detail by van den Berg et al. [11], since we excluded the crossbred cows from the original data. The dataset consisted of WGS and high-density (HD) genotype data and daughter yield deviations (DYD; in the case of bulls) or yield deviations (YD; in the case of cows) for 35,549 bulls and cows. The DYD and YD were available for five traits: kg of milk, kg of fat, kg of protein, fat percentage, and protein percentage. The dataset was divided into a training or reference and a validation population as shown in Table 1. Animals in the reference population that had sons in the validation population and daughters of validation bulls were removed from the dataset, in order to reduce the links between the reference and validation sets. The validation population consisted of all AR cows, and H bulls and J bulls born after 2005. The reference population contained H bulls born before 2005, all H cows, J bulls born before 2005 and all J cows. The reference population contained no AR animals, i.e. predicted AR phenotypes were entirely based on across-breed genomic prediction. Animals were either directly genotyped with the Illumina 800 K BovineHD bead chip (HD), or first genotyped with the Illumina BovineSNP50K chip [12] or a lower density SNP chip, and subsequently imputed to HD. All individuals were imputed to WGS using a reference population of H, J and AR bulls and cows from Run 5 of the 1000 bulls genome project and the UMD3.1 reference sequence [3]. FImpute [5] was used for genotype imputation. After filtering out variants with a minor allele frequency (MAF) lower than 0.002 and LD pruning (r^2^ > 0.9) using PLINK [13], 4,809,520 variants were retained for the analysis. Genotypes were phased using Eagle2 [14].

**Table 1:** Numbers of reference and validation animals per breed and sex

	Data	Number of reference animals	Number of validation animals
Holstein bulls	DYD	3124^a^	826^b^
Holstein cows	YD	22,868	0
Jersey bulls	DYD	787^a^	221^b^
Jersey cows	YD	6144	0
Australian Red cows	YD	0	1579
Total		32,923	2626

DYD: daughter yield deviation; YD: yield deviation

^a^Born before 2005 and ^b^born after 2005

### Statistical model of Bayes GC

The phenotypes (YD and DYD) are modelled as the sum of fixed breed*sex effects, a polygenic genetic value fitted by a GBLUP term, and the effects of SNPs fitted by a Bayes C term [15], resulting in the model:

1 $$\mathbf{y}=\mathbf{Fb}+\mathbf{u}+\sum_{\text{i}=1}^{4,809,520}\text{I}_{\text{i}}\bf{x}_{\bf{i}}\text{s}_{\text{i}}+\mathbf{e},$$

where $\mathbf{F}$ denotes the design matrix of the fixed breed*sex effect ($\mathbf{b}$), $\mathbf{u}$ is a $N\times 1$ vector of polygenic effects with $Var\left(\mathbf{u}\right)=\mathbf{G}{\upsigma }_{\mathrm{u}}^{2}$, where $\mathbf{G}$ is the genomic relationship matrix, ${\upsigma }_{\mathrm{u}}^{2}$ is the polygenic variance; ${\mathrm{I}}_{\mathrm{i}}=1$ if the SNP is included in the model and ${\mathrm{I}}_{\mathrm{i}}=0$ otherwise; ${\mathbf{x}}_{{\varvec{i}}}$ is a (35,688 $\times$ 1) vector of genotypes for SNP $i$; ${\mathrm{s}}_{i}$ is the effect of SNP $i$ with prior distribution ${\mathrm{s}}_{i}\sim N(0,{\upsigma }_{\mathrm{s}}^{2}$), and ${\upsigma }_{\mathrm{s}}^{2}$ is the variance of the SNP effects. Setting up a WGS-based genomic relationship matrix is computationally costly, thus here, we used the genotypes from the HD SNP chip to set up $\mathbf{G}$ using VanRaden’s Method 2 [16] that was applied across breeds using a single reference allele frequency for each SNP; and $\mathbf{e}$ denotes a vector of residuals with $Var\left(\mathbf{e}\right)=\mathbf{R}{\upsigma }_{\mathrm{e}}^{2}$, where ${\upsigma }_{\mathrm{e}}^{2}$ is the residual variance and ${\mathbf{R}}^{-1}$ is a diagonal matrix with weights of the records. Missing records are accommodated by sampling them within the MCMC scheme.

Model (1) is prone to over-parametrization since both the GBLUP and the Bayes C term alone can explain all the genetic variance. In addition to fitting all SNPs by the GBLUP term, we want the Bayes C term to fit the top $\pi$*100% SNPs with the largest effect individually and thereby improve prediction accuracy, where $\pi$ is the prior probability that a SNP has a large effect (${\mathrm{I}}_{\mathrm{i}}=1$), i.e. a priori Prob(${\mathrm{I}}_{\mathrm{i}}=1$) = $\pi$. To reduce over-parameterization, we choose to fit ~ 2500 SNPs with large effects, and estimate the average variance explained by these top SNPs, ${\upsigma }_{\mathrm{s}}^{2}$. Our choice of ~ 2500 large effect SNPs agrees with Wood et al. [17], who found that 2000 to 3700 SNPs explained 21 to 24% of the variance in human height, i.e. by fitting ~ 2500 SNPs, our aim was to explain ~ 20% of the genetic variance. Hence, a fixed $\pi$ value of ~ 0.0005 was used.

### Fitting the models by MCMC

The fixed effects are sampled by Gibbs sampling. The effect of (breed*sex)_*i*_ is sampled within each MCMC cycle from its conditional posterior distribution [18]:

$${b}_{i}\sim N(\frac{{\mathbf{F}}_{{\varvec{i}}}\mathrm{^{\prime}}{\mathbf{R}}^{-1}{\mathbf{y}}^{*}}{{\mathbf{F}}_{{\varvec{i}}}\mathrm{^{\prime}}{\mathbf{R}}^{-1}{\mathbf{F}}_{{\varvec{i}}}};\frac{{\upsigma }_{\mathrm{e}}^{2}}{{\mathbf{F}}_{{\varvec{i}}}\mathrm{^{\prime}}{\mathbf{R}}^{-1}{\mathbf{F}}_{{\varvec{i}}}}),$$

where ${\mathbf{y}}^{\mathbf{*}}$ denotes the vector of data corrected for all other (genetic) effects in the model, and ${\mathbf{F}}_{{\varvec{i}}}$ denotes the $i$-th column of the design matrix $\mathbf{F}$.

#### Polygenic term

The polygenic effects were updated in one block by a Gibbs sampling step from its conditional posterior distribution [18]:

$$\mathbf{u}\sim \mathrm{N}[{\left({\mathbf{R}}^{-1}+{\upkappa \mathbf{G}}^{-1}\right)}^{-1}{\mathbf{R}}^{-1}{\mathbf{y}}^{*};{\left({\mathbf{R}}^{-1}+{\upkappa \mathbf{G}}^{-1}\right)}^{-1}{\upsigma }_{\mathrm{e}}^{2}],$$

where ${\mathbf{y}}^{\mathbf{*}}$ denotes the vector of data corrected for all other effects in the model, and $\upkappa ={\upsigma }_{\mathrm{e}}^{2}/{\upsigma }_{\mathrm{u}}^{2}$. This block sampling requires the inverse of the coefficient matrix $\left({\mathbf{R}}^{-1}+{\upkappa \mathbf{G}}^{-1}\right)$, which is number of animals by number of animals, and $\upkappa ={\upsigma }_{\mathrm{e}}^{2}/{\upsigma }_{\mathrm{u}}^{2}$ may vary from one cycle to the next, since ${\upsigma }_{\mathrm{u}}^{2}$ is re-sampled every cycle. To reduce the amount of computations per MCMC cycle, we diagonalize the coefficient matrix following the approach of de los Campos et al. [19]:

$$\left({\mathbf{R}}^{-1}+{\upkappa \mathbf{G}}^{-1}\right)={\mathbf{R}}^{-1/2}\left(\mathbf{I}+{\upkappa {\mathbf{R}}^{1/2}\mathbf{G}}^{-1}{\mathbf{R}}^{1/2}\right){\mathbf{R}}^{-1/2},$$

where ${\mathbf{R}}^{-1/2}$ is a diagonal matrix containing the square-root of the elements of ${\mathbf{R}}^{-1}$. Next, the eigen-decomposition of ${{\mathbf{R}}^{1/2}\mathbf{G}}^{-1}{\mathbf{R}}^{1/2}$ is obtained, i.e.:

$${{\mathbf{R}}^{1/2}\mathbf{G}}^{-1}{\mathbf{R}}^{1/2}=\mathbf{E}\mathrm{^{\prime}}\mathbf{D}\mathbf{E},$$

where $\mathbf{E}$ is a matrix of orthonormal eigenvectors and $\mathbf{D}$ is a diagonal matrix of eigenvalues. Since $\mathbf{E}\mathbf{^\prime}\mathbf{E}=\mathbf{I}$, it follows that:

$$\left({\mathbf{R}}^{-1}+{\upkappa \mathbf{G}}^{-1}\right)={\mathbf{R}}^{-1/2}\left(\mathbf{E}\mathrm{^{\prime}}\mathbf{E}+\upkappa \mathbf{E}\mathrm{^{\prime}}\mathbf{D}\mathbf{E}\right){\mathbf{R}}^{-1/2}={\mathbf{R}}^{-1/2}\mathbf{E}\mathrm{^{\prime}}\left(\mathbf{I}+\upkappa \mathbf{D}\right)\mathbf{E}{\mathbf{R}}^{-1/2},$$

and its inverse is:

$${\left({\mathbf{R}}^{-1}+{\upkappa \mathbf{G}}^{-1}\right)}^{-1}={\mathbf{R}}^{1/2}\mathbf{E}\mathrm{^{\prime}}{\left(\mathbf{I}+{\varvec{\upkappa}}\mathbf{D}\right)}^{-1}\mathbf{E}{\mathbf{R}}^{1/2},$$

where the inverse of $\left(\mathbf{I}+\upkappa \mathbf{D}\right)$ is easily obtained since it is a diagonal matrix (even when $\upkappa$ varies). Thus, the calculation of ${\left({\mathbf{R}}^{-1}+{\upkappa \mathbf{G}}^{-1}\right)}^{-1}{\mathbf{R}}^{-1}{\mathbf{y}}^{\mathbf{*}}$ (as shown above)**,** requires the calculation of ${\mathbf{R}}^{1/2}\mathbf{E}{^{\prime}}{\left(\mathbf{I}+\upkappa \mathbf{D}\right)}^{-1}\mathbf{E}{\mathbf{R}}^{1/2}{(\mathbf{R}}^{-1}{\mathbf{y}}^{\mathbf{*}})$**,** where ${\mathbf{R}}^{-1}{\mathbf{y}}^{\mathbf{*}}$ is a vector of right-hand-sides (RHS). These calculations are performed by multiplying this RHS vector with each of the required matrices starting with the right-most (${\mathbf{R}}^{1/2}$), followed by multiplying the resulting vector with $\mathbf{E}$, and working our way towards the left-most matrix. In this way, only matrix times vector multiplications are required which are of the order of ${\mathrm{N}}^{2}$ operations (or $\mathrm{N}$ operations if the matrix is diagonal). For comparison, matrix inversion requires of the order of ${\mathrm{N}}^{3}$ operations. Although, the calculation of the eigen-decomposition of ${{\mathbf{R}}^{1/2}\mathbf{G}}^{-1}{\mathbf{R}}^{1/2}$ is computer intensive when the number of animals is large, it is performed only once before starting the MCMC sampling.

#### Sampling of SNP effects

Within any cycle c of the MCMC algorithm, millions of SNPs are not in the model and almost all do not remain in the model when moving to the next cycle c + 1, i.e. their evaluation does not result in a move of the MCMC chain. Of course, some of these evaluations of SNPs do result in a SNP move, i.e. a change of the effect of a SNP. In order to direct computer efforts towards SNPs for which the estimates of their effects are expected to change, we will update in cycle c the SNPs that currently have an effect of zero (i.e. are not in the model) with a reduced probability of ${v}_{i}$ implemented by a Metropolis–Hastings (MH) step. The updating probabilities of the SNPs (${v}_{i}$) followed a geometric distribution:

$${v}_{i}={(1-\rho )}^{{r}_{i}},$$

where ${r}_{i}$ is the ranking (from high to low) of SNP $i$ based on its log-posterior probability of being fitted (${\theta }_{i}$; from (3) below); and $\rho$ was chosen such that the SNP with the lowest ${\theta }_{i}$ would be expected to be evaluated 100 times (i.e. ${(1-\rho )}^{\mathrm{4,809,520}}*C=100$, where $C$ is the total number of MCMC cycles).

The ${\theta }_{i}$-values (from (3); see below), that were used for the ranking of the SNPs to calculate ${v}_{i}$, were calculated during the first cycle of the MCMC chain, and none of the SNPs was fitted during this first cycle in order to evaluate ${\theta }_{i}$ of SNP $i$ when no other SNP was fitted (similar to a genome-wide association study (GWAS) where P-values are calculated for each SNP in turn). However, the records had been corrected for fixed effects and the GBLUP term $\mathbf{u}$. Hence, SNPs with high ${\theta }_{i}$-values have an increased probability of being evaluated. This updating probability was constant from the first MCMC cycle till the last one, and the updating probability ${v}_{i}$ decreases with the ranking of the SNPs following a geometric distribution. A comparable prioritization of SNPs is implemented in BLSMM [9], but BLSMM samples the SNP to be evaluated using a mixture of a uniform and a geometric distribution, whereas here the probability of skipping a non-fitted SNP follows a geometric distribution.

If SNP $i$ is updated, we need the log posterior probability of not fitting SNP $i$ in the model [20]:

2 $${\theta }_{0}=-\frac{1}{2{\upsigma}_{\text{e}}^{2}}{\mathbf{{y}^{*}}}{^{\prime}}{\mathbf{ {R}^{-1} {y}^{*}} } -\frac{1}{2}nlog\left({\upsigma }_{\text{e}}^{2}\right)-\frac{1}{2}{\text{log}}\left(\left|\mathbf{R}\right|\right)+{\text{log}}\left(1-\pi \right)={L}_{0}+{\text{log}}\left(1-\pi \right),$$

where $n$ is the number of records; and ${L}_{0}$ is the log-likelihood of no SNP in the model. The log posterior probability of fitting SNP $i$ in the model is [20]:

3 $${\theta }_{i}={L}_{0}+\frac{1}{2{\upsigma }_{\mathrm{e}}^{2}}\frac{{({\mathbf{x}}_{{\varvec{i}}}{^{\prime}}{\mathbf{R}}^{-1}{\mathbf{y}}^{\mathbf{*}})}^{2}}{{\mathbf{x}}_{{\varvec{i}}}{^{\prime}}{\mathbf{R}}^{-1}{\mathbf{x}}_{{\varvec{i}}}+\lambda }+\frac{1}{2}\mathrm{log}\left(\lambda \right)-\frac{1}{2}\mathrm{log}({\mathbf{x}}_{\mathbf{i}}{^{\prime}}{\mathbf{R}}^{-1}{\mathbf{x}}_{{\varvec{i}}}+\lambda )+\mathrm{log}(\pi ),$$

where ${L}_{0}$ is from (2), and $\lambda ={\upsigma }_{\mathrm{e}}^{2}/{\upsigma }_{\mathrm{s}}^{2}$, and ${\upsigma }_{\mathrm{s}}^{2}$ is the variance of the SNP effect which is assumed normally distributed if there is an effect (as in Bayes C [15]).

If SNP $i$ is currently not in the model (${I}_{i}=0$), we propose that it enters the model with probability ${v}_{i}$, and with probability (1-${v}_{i}$) the SNP remains with (${I}_{i}=0$), i.e. the evaluation of the SNP is skipped. The updating of SNP $i$ involves a MH-step: we accept the proposal of the SNP entering the model with a MH-acceptance-probability of:

$${\alpha }_{{\mathrm{I}}_{i}=0\to 1}=\mathrm{min}(1,\frac{\mathrm{exp}\left({\theta }_{i}\right)}{\mathrm{exp}\left({\theta }_{0}\right){v}_{i}}).$$

Alternatively, if SNP $i$ is currently in the model, we propose with a probability of 1 that it moves out of the model, and accept this proposal with an MH-acceptance-probability of:

$${\alpha }_{{\mathrm{I}}_{i}=1\to 0}=\mathrm{min}(1,\frac{\mathrm{exp}\left({\theta }_{0}\right){v}_{i}}{\mathrm{exp}\left({\theta }_{i}\right)}).$$

In these acceptance probabilities, the term ${v}_{i}$ corrects for the fact that the evaluation of SNPs that are not in the model is skipped with a probability of 1-${v}_{i}$.

If SNP $i$ remains/enters in the model (${\mathrm{I}}_{i}=1$), we continue updating its effect by sampling an effect for SNP $i$ from its conditional posterior distribution [18]:

$${s}_{i}\sim N(\frac{{\mathbf{x}}_{{\varvec{i}}}\mathrm{^{\prime}}{\mathbf{R}}^{-1}{\mathbf{y}}^{*}}{{\mathbf{x}}_{{\varvec{i}}}\mathrm{^{\prime}}{\mathbf{R}}^{-1}{\mathbf{x}}_{{\varvec{i}}}+\lambda };\frac{{\sigma }_{e}^{2}}{{\mathbf{x}}_{{\varvec{i}}}\mathrm{^{\prime}}{\mathbf{R}}^{-1}{\mathbf{x}}_{{\varvec{i}}}+\lambda }).$$

Finally, we correct the data ${\mathbf{y}}^{\mathbf{*}}$ for the new SNP effect, and continue with the next SNP $i$+1. If SNP $i$ is not in the model (${\mathrm{I}}_{i}=0$), correction of the data corrected for all other effects in the model (${\mathbf{y}}^{\mathbf{*}}$) is not needed, which saves computer time.

### Sampling of ${{\varvec{\upsigma}}}_{\mathbf{s}}^{2}$ and ${{\varvec{\upsigma}}}_{\mathbf{u}}^{2}$

The variance of the SNPs with large effects and that of the polygenic effects are sampled in the same manner, in order to unbiasedly balance these two variances against each other. Assuming a flat prior distribution, ${\upsigma }_{\mathrm{s}}^{2}$ is sampled from its conditional posterior distribution [18]:

$${\upsigma }_{\mathrm{s}}^{2}\sim {\mathbf{s}}^{{^{\prime}}}\mathbf{s}/{\upchi }_{(\sum {\mathrm{I}}_{i}-2)}^{2},$$

where ${\upchi }_{(\sum {\mathrm{I}}_{i}-2)}^{2}$ denotes a sample from the chi-squared distribution with the number of fitted SNPs minus 2 degrees of freedom; $\mathbf{s}$ is a ($\sum {\mathrm{I}}_{i}\times 1$) vector of current estimates of SNP effects.

Similarly, the polygenic variance ${\upsigma }_{\mathrm{u}}^{2}$ is sampled from its conditional posterior distribution [18]:

$${\upsigma }_{\mathrm{u}}^{2}\sim {\mathbf{u}}^{{^{\prime}}}{\mathbf{G}}^{-1}\mathbf{u}/{\chi }_{(N-2)}^{2},$$

where $\mathbf{u}$ is a $N\times 1$ vector containing the current estimate of the polygenic effects. The error variance ${\upsigma }_{\mathrm{e}}^{2}$ was not updated and thus assumed known, e.g. from a larger dataset containing also ungenotyped individuals. The number of hyper-parameters, such as ${\upsigma }_{\mathrm{e}}^{2}$, that needed to be estimated, was kept as small as possible in order to keep the number of required MCMC cycles as small as possible.

### Computational efficiency

Storing of 4,809,520 SNP genotypes on 35,688 individuals in single precision, which would allow storing centered/scaled genotypes (4 bytes per genotype) would require 687 Gbytes, which exceeds the RAM of most computers. In PLINK [13], genotypes are stored bitwise in binary files (.bed files). Binary storage uses 2 bits per genotype, i.e. 4 genotypes per byte. We used a similar approach and used 2 bits to store the genotypes codes 0 (homozygote reference allele), 1 (heterozygote), or 2 (homozygote alternative allele), i.e. bitwise ‘00’, ‘01’, and ‘10’, respectively. The 2 bits were read from a regular integer number by the intrinsic Fortran90 function *ibits*. This reduced the storage requirements of the genotypes by 16-fold at the computational cost of calling the *ibits*-function whenever genotypes were needed. In this way, all ($\mathrm{4,809,520}*\mathrm{35,688}$) genotypes could be stored within 43 Gbytes, i.e. within the RAM of a large laptop.

A drawback of the binary storage of genotypes is that the stored genotypes are not centralized, whereas in genomic prediction random regression is typically on centralized genotypes (e.g. [16]). Otherwise the estimates of the SNP effects also affect the mean breeding value of the population, which is commonly assumed to be 0 (e.g. [21]). Changes in the population mean may also slow down the convergence rate of the MCMC chain. Obviously, we could centralize the genotypes after obtaining them from binary storage, but this is computationally costly since it needs to be repeated for every MCMC cycle.

The centralized genotypes are used to calculate right-hand-side ${\mathbf{x}}_{{\varvec{i}}}{^{\prime}}{{\mathbf{R}}^{-1}\mathbf{y}}^{\mathbf{*}}$ and the SNP’s contribution to the diagonal of the mixed model equations: ${\mathbf{x}}_{{\varvec{i}}}{^{\prime}}{\mathbf{R}}^{-1}{\mathbf{x}}_{{\varvec{i}}}$. Let ${\stackrel{\sim }{\mathbf{x}}}_{{\varvec{i}}}$ denote a vector of uncentralised genotypes for SNP $i$ containing the codes 0, 1, or 2 with a weighted mean value of ${\stackrel{-}{x}}_{i}={\mathbf{1}{^{\prime}}\mathbf{R}}^{-1}{\mathbf{x}}_{{\varvec{i}}}/{\mathbf{1}{^{\prime}}\mathbf{R}}^{-1}\mathbf{1}$, where weighing is by the weights of the records. The weighted mean of the genotypes needs to be calculated only once. Then, the centralized genotypes are ${\mathbf{x}}_{{\varvec{i}}}={\stackrel{\sim }{\mathbf{x}}}_{{\varvec{i}}}-1{\stackrel{-}{\mathbf{x}}}_{{\varvec{i}}}$, and the right-hand-side is:

$${\mathbf{x}}_{{\varvec{i}}}{^{\prime}}{{\mathbf{R}}^{-1}\mathbf{y}}^{*}={\stackrel{\sim }{\mathbf{x}}}_{{\varvec{i}}}{^{\prime}}{{\mathbf{R}}^{-1}\mathbf{y}}^{*}-{\stackrel{-}{x}}_{i}\mathbf{1}\mathrm{^{\prime}}{{\mathbf{R}}^{-1}\mathbf{y}}^{*},$$

where $\mathbf{1}{^{\prime}}{{\mathbf{R}}^{-1}\mathbf{y}}^{\mathbf{*}}$ is the weighted sum of the corrected records, ${\mathbf{y}}^{\mathbf{*}}$. The contribution of SNP $i$ to the diagonal of the mixed model equations can be rewritten as:

$${(\stackrel{\sim }{\mathbf{x}}}_{{\varvec{i}}}-\mathbf{1}{\stackrel{-}{\mathbf{x}}}_{{\varvec{i}}})\boldsymbol{^{\prime}}{\mathbf{R}}^{-1}{(\stackrel{\sim }{\mathbf{x}}}_{{\varvec{i}}}-\mathbf{1}{\stackrel{-}{\mathbf{x}}}_{\mathbf{i}})$$

$$={\stackrel{\sim }{\mathbf{x}}}_{{\varvec{i}}}\boldsymbol{^{\prime}}{\mathbf{R}}^{-1}{\stackrel{\sim }{\mathbf{x}}}_{{\varvec{i}}}-2{\stackrel{\sim }{\mathbf{x}}}_{{\varvec{i}}}\boldsymbol{^{\prime}}{\mathbf{R}}^{-1}{\mathbf{1}\stackrel{-}{x}}_{i}+\mathbf{1}\boldsymbol{^{\prime}}{\mathbf{R}}^{-1}\mathbf{1}{\stackrel{-}{x}}_{{\varvec{i}}}^{2}$$

$${=\,\stackrel{\sim }{\mathbf{x}}}_{{\varvec{i}}}\mathrm{^{\prime}}{\mathbf{R}}^{-1}{\stackrel{\sim }{\mathbf{x}}}_{{\varvec{i}}}-(\mathbf{1}{\mathrm{^{\prime}}\mathbf{R}}^{-1}\mathbf{1}){\stackrel{-}{x}}_{i}.$$

Thus, the right-hand-side and the contribution to the diagonal of the mixed model equations for the centralized genotypes could be calculated from their uncentralised counterparts within every MCMC cycle, by calculating the weighted mean of the genotypes, ${\stackrel{-}{\mathbf{x}}}_{{\varvec{i}}}$, and the sum of the weights $\mathbf{1}{^{\prime}}{\mathbf{R}}^{-1}\mathbf{1}$ before starting the MCMC calculations.

Modern computers can run many processes simultaneously. In case of MCMC sampling, this suggests running many short MCMC chains simultaneously instead of a single long one. The latter also benefits convergence diagnostics: the variability of the MCMC outcomes across the chains are indicative of the standard errors due to MCMC sampling [22]. Running multiple MCMC chains simultaneously could be achieved by running multiple instances of a single threaded MCMC program where each program runs one of the chains. However, in this setting, every chain will require a lot of computer memory since all genotypes need to be stored for each of the chains. Memory limitations will limit the number of chains that can be run simultaneously.

To make more efficient use of computational resources, we developed a parallel Fortran90 computer program that simultaneously ran multiple MCMC chains but kept only a single genotype matrix in RAM storage. The latter was achieved by setting up a parallel loop that runs the MCMC cycling loop multiple times using the OpenMP directive. Moreover, we assumed that some hyper-parameters such as the error variance (${\upsigma }_{\mathrm{e}}^{2}$) and prior probabilities of SNP effects ($\pi$) are known, which reduced the required length of the MCMC chain.

In all MCMC chains, 10,000 MCMC cycles were performed, of which the first 2000 were discarded as burn-in. Bayes GC genomic breeding value estimates (GEBV) were obtained from.

$$\mathrm{GEBV}=average[\mathbf{u}+\sum_{i=1}^{\mathrm{4,809,520}}{\mathrm{I}}_{i}{\mathbf{x}}_{{\varvec{i}}}{s}_{i}],$$

where averaging is across 8000 non-burn-in cycles and across 10 parallel chains. GEBV using GBLUP were obtained by using the Bayes GC software but setting the prior probability of including SNPs in the model to $\pi=0$, which implies that also all ${\mathrm{I}}_{i}=0$, and only the polygenic component $\mathbf{u}$ remained. Convergence was checked by comparing the GEBV of 10 replicated MCMC chains, and the correlation between the GEBV from different chains was always higher than 0.999. For estimates of individual SNP effects, this figure was on average 0.895, suggesting that more cycles are needed to obtain converged estimates for individual SNP effects as for GEBV. The Bayes GC software is available from the authors upon request.

### Detecting QTL using Bayes GC

Bayes GC can be used to map causal variants to regions (we used 250-kb regions) and to individual sequence variants. First, the importance of a region for harboring genetic effects was quantified by the variance of the local GEBV for this region as calculated based on the Bayes C term in model (1), i.e. excluding the GBLUP term which is considered to explain an equal amount of variance for all positions. Second, the mapping precision was further increased by examining the posterior probabilities of the SNPs in the 250-kb region, which are the proportion of MCMC cycles past burn-in where the SNP was included in the model (${\mathrm{I}}_{i}=1$).

## Results

### QTL mapping

**Fig. 1:**
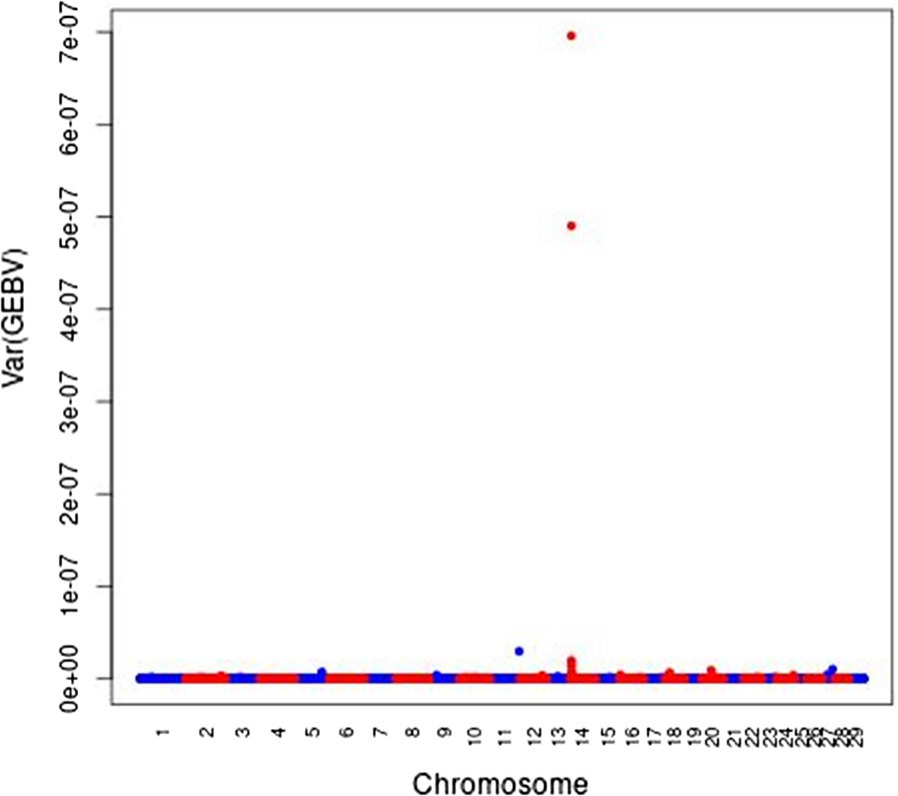
Manhattan plots of the variance of the local GEBV within 250-kb regions for fat percentage

Figure 1 shows the Manhattan plot of the variances of local GEBV for fat percentage calculated in 250-kb regions across the genome, as an indicator for the genetic variance contained in the regions [23], which indicates whether the region contains important QTL. We compare our results on fat percentage to a recent meta-analysis of eight cattle breeds by van den Berg et al. [24], which included the data from the Holstein and Jersey individuals used here. As expected, QTL signals are dominated by the *DGAT1* gene, which is located at the beginning of *Bos taurus* chromosome (BTA)14 [25]. This is also the case for the other traits, and their Manhattan plots are shown in see Additional file 1: Figures S1 to S4. Many less strong signals occur mainly on BTA2, 5, 6, 11, 16 and 20 on which QTL were reported by several GWAS studies (e.g. [26–29]).

**Fig. 2:**
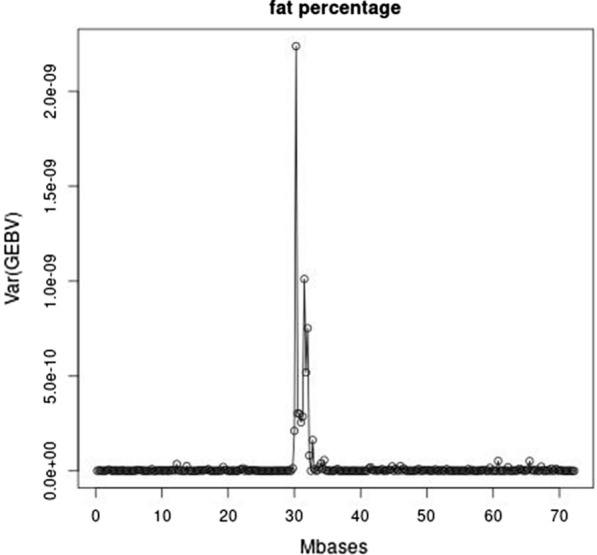
Manhattan plot of the variance of the local GEBV within 250-kb regions for fat percentage on BTA20

The meta-analysis of van den Berg et al. [24] detected 80 significant COJO-SNPs (conditional and joint analysis as implemented in GCTA [30]) for fat percentage. Many of these SNPs are not present in our current data due to differences in variant selection criteria and quality control, when processing the sequence data. The top 10 250-kb-regions with the largest variance of local GEBV for fat percentage contained six of these COJO-SNPs: two at the beginning of BTA14, and one region on each of BTA2, 5, 11, and 20. In addition, the top 10 250-kb regions contained four more regions that were near the aforementioned regions with COJO-SNPs on BTA14. As an example, Fig. 2 shows the variance of local GEBV for fat percentage at BTA20. BTA20 seems to harbor two fat percentage QTL close to each other between 30 and 35 Mb. The second peak is not in the top 10 250-kb-regions but is sufficiently high to be within the top 20 regions. Detailed maps of the variance of local GEBV for the other QTL in the top 10 250-kb regions are shown in see Additional file 2: Figures S5 to S8 for BTA2, 5, 11, and 14, respectively.

**Fig. 3:**
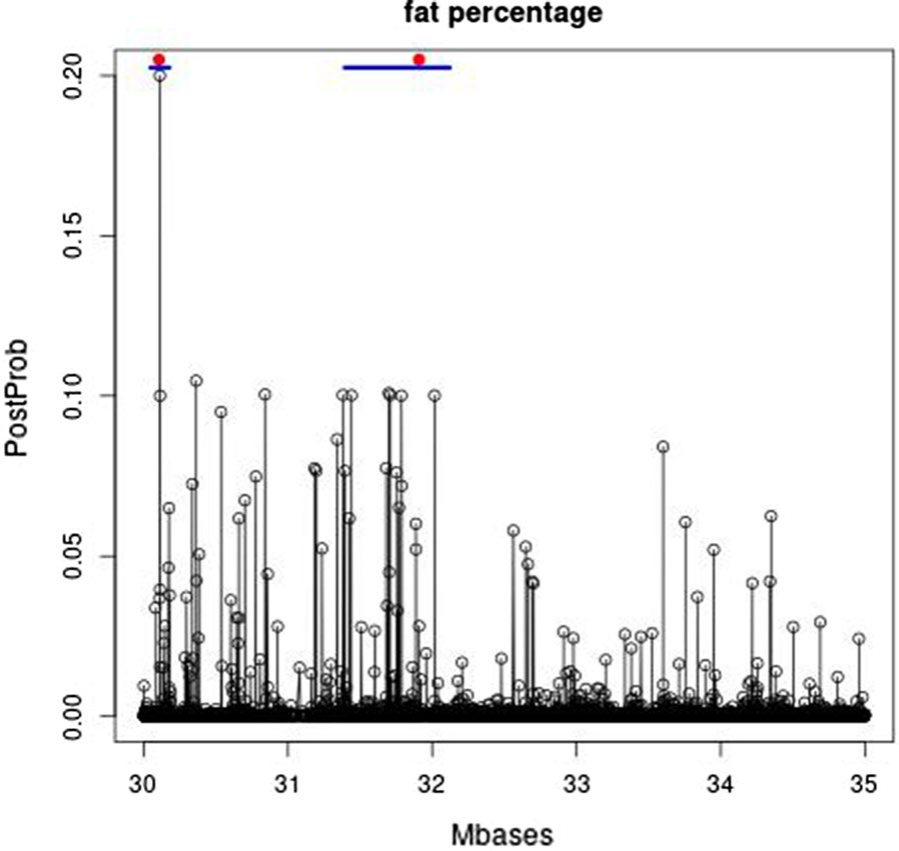
Fine-scale map of the posterior probabilities of the SNPs for affecting fat percentage in the region between 30 and 35 Mb on BTA20. The blue bar denotes the 95% credibility interval for the QTL, and the red dot the position of the COJO SNP detected by [23]

To further fine-map the QTL on BTA20, Fig. 3 shows the posterior probability of the SNPs in the region between 30 and 35 Mb. The highest posterior probabilities of the SNPs within each of the two 250-kb regions that are in the top 20 were at 30.112083 Mb and 31.786449 Mb for the first and second peak, respectively. The corresponding positions of the COJO-SNPs detected by [24] were at 30.106314 and 31.909478 Mb, respectively, where the latter variant corresponds to the F279Y mutation in the *GHR* gene, which has major effects on milk yield and composition [31].

For each of the top-SNPs, a 95% posterior credibility interval was constructed by: (1) identifying within each MCMC cycle (excluding burn-in cycles) which SNP currently fitted in the model was nearest to the top-SNP in the 250-kb region (i.e. nearest to positions 30.112083 and 31.786449, respectively) under the restriction that the nearest SNP was less than 500 SNPs away (i.e. a SNP fitted more than 500 positions away is assumed to point to a different QTL, and is thus ignored); and (2) trimming-off the 2.5% SNPs that are the furthest away from either side of the region. The 95% credibility interval was between 30.046906 and 30.177482 Mb for the first top-SNP, and between 31.394136 and 32.121047 for the second top-SNP. Both 95%-credibility intervals contained the corresponding COJO SNPs detected by [24]. Posterior probabilities together with their 95% credibility intervals are shown for the QTL at BTA2, 5, 11, and 14 in see Additional file 3: Figures S9 to S12, respectively. All these 95% credibility intervals included their corresponding COJO SNPs. At the beginning of BTA14, there are several causal variants that explain the QTL signals [24], and this hampers the positioning of the QTL due to carry-over effects of other QTL. The K232A mutation in the *DGAT1* gene [25] seems to have been captured by two high peaks at the beginning of BTA14. There are several causal variants at the beginning of BTA14, which explain the QTL signals [24], and hamper the accurate positioning of the QTL. We set up the 95% credibility interval surrounding the second peak, since this peak was within the 250-kb region with the highest variance of local GEBV. This 95% credibility interval included both the COJO SNP detected by [24] and the K232A mutation in *DGAT1*. There were two more COJO SNPs within the first four Mb of BTA14, but their positions were not clear from the posterior probabilities provided in Additional file 3 due to interferences of QTL signals.

When extending the top 10 to the top 20 250-kb regions with largest variance of local GEBV, three more COJO SNPs [24] were detected. One QTL on BTA20 as shown in Fig. 3, and one more at the beginning of BTA14. A QTL at the beginning of BTA16 was detected at 1.566222 Mb. Beyond the top 20, relatively few additional COJO SNPs were detected. E.g. the top 50 250-kb regions with largest variances of local GEBV contained only one additional COJO SNP compared to the top 20.

**Fig. 4:**
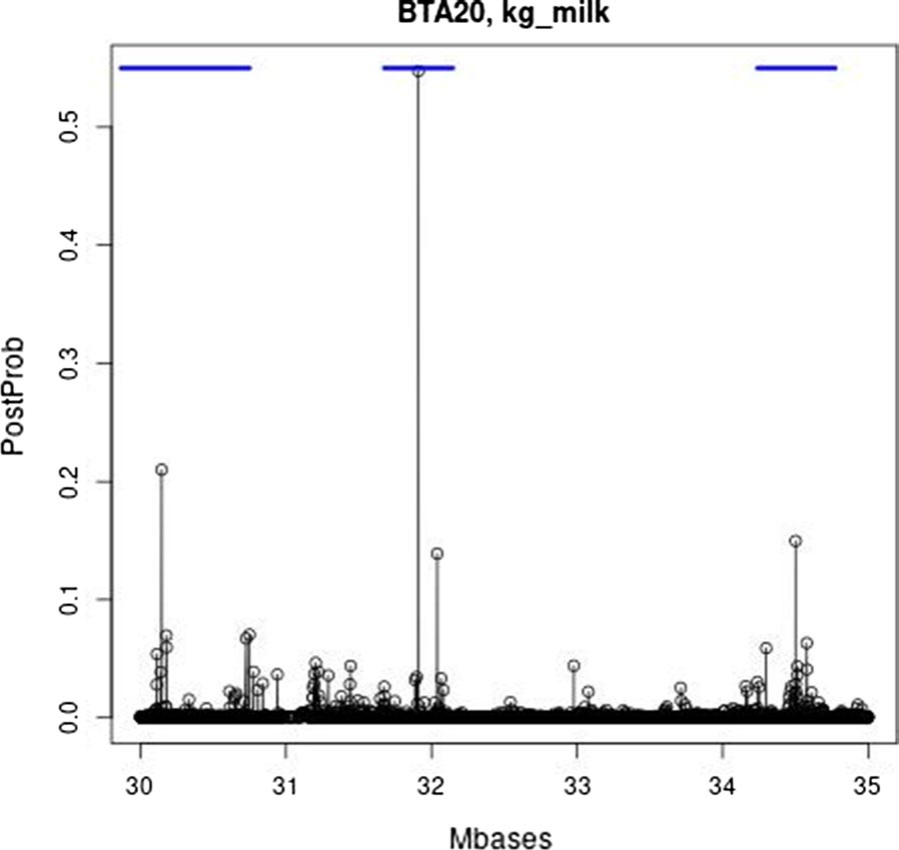
Fine-scale map of the posterior probabilities of the SNPs for affecting milk production in the region between 30 and 35 Mb on BTA20. The blue bar denotes the 95% credibility interval for the QTL

The QTL signals within the region between 30 and 35 Mb on BTA20 are more clearly depicted by considering milk production instead of fat percentage (Fig. 4). The posterior probabilities of SNPs for milk production indicate three QTL within the 30–35 Mb region. The 250-kb regions to which these QTL belong are all within the top 20 for the variances of local GEBV for milk production. Hence, the previously identified QTL for fat percentage seemed also to affect milk production, and were positioned at 30.145126 and 31.909478 Mb. The former SNP is within 39 kb from the COJO-SNP detected by van den Berg et al. [24], and the latter is exactly at the F279Y mutation in *GHR* [31]. Furthermore, a new, additional QTL was found to affect milk production (see Fig. 4) at position 34.501126 Mb. To the best of our knowledge, this QTL has not been reported before.

The QTL detected in the top 10 250-kb regions for fat and protein yield are provided together with their 95% credibility intervals in see Additional file 4: Tables S1 and S2, respectively. For fat yield, QTL were detected on BTA5, 14, 19, 23, 24, 26 and 27. For protein yield, QTL were detected on BTA4, 5, 6, 9, 11, 14. The 95% credibility intervals for the QTL on BTA5 and 14 overlap. Only nine QTL positions are provided for protein yield since the 10th top-SNP had a posterior probability lower than 0.01, which hardly exceeds the prior probability. Some of the SNPs with the highest posterior probability were in more than 95% of the MCMC cycles included in the model, which implies that their 95% credibility interval contains only one SNP. If the credibility interval had been based on the cumulative posterior probabilities of the SNPs in the interval, then also only one SNP would pass the posterior probability threshold of 95% and the interval would be the same as shown in see Additional file 4: Tables S1 and S2, respectively. If the causative mutation is not included the genotype data, this single SNP interval merely points to the SNP which is most strongly associated with the causative mutation. The latter would thus not reside in the interval, which contains only one (most associated) SNP.

### Genomic predictions

Table 2 shows the accuracies of prediction measured by the correlation between GEBV and DYD/YD in the validation sets. For AR cows these correlations are substantially lower since their YD have lower accuracy (correlation between YD and true breeding value) than the DYD of H and J bulls, and because their GEBV are entirely based on across-breed predictions. AR cows had no within-breed prediction, since there were no AR reference animals. Prediction accuracies of J bulls were relatively high when compared to H bulls, which is probably due to their smaller effective population size whereas Australian Holsteins include Holstein genes from all over the world. The $\mathbf{G}$ matrices were built using HD SNP-chip data, and thus the GBLUP methods used only HD SNP-chip data. When moving from within-breed to between-breed GBLUP, the prediction accuracies of the H bulls and J bulls improved by less than 0.04. When progressing from GBLUP to Bayes GC across-breed predictions, prediction accuracies of H bulls further improved by up 0.02. For J bulls, these improvements were somewhat larger, i.e. by up to a factor of 0.05. The AR cows obtained an across-breed based genomic prediction accuracy of 0.17 to 0.25 using GBLUP. When moving to Bayes GC, the prediction accuracies of AR cows increased by a factor of 0.09 to 0.29 for kg milk, kg fat, kg protein and fat percentage. The prediction accuracy of AR cows for protein percentage almost doubled, but this seemed to be due to a remarkably low accuracy of the across-breed GBLUP prediction (especially in view of the high heritability of protein percentage).

**Table 2:** Correlation between GEBV and (D)YD^a^ for kg milk, kg fat, kg protein, fat percentage and protein percentage using within-breed (WB) and across-breed (AB) GBLUP, and across-breed Bayes GC predictions

	N^b^	GBLUP(WB)	GBLUP(AB)	BayesGC	Accuracy (D)YD
kg milk (variance due to SNPs 15%; posterior probability = 0.00048) ^c^					
Holstein bulls	826	0.713	0.714	0.729	0.970
Jersey bulls	221	0.617	0.643	0.674	0.964
Australian Red cows	1579	–	0.23	0.263	0.469
kg fat (variance due to SNPs 25%; posterior probability = 0.00049) ^c^					
Holstein bulls	826	0.655	0.658	0.669	0.954
Jersey bulls	221	0.699	0.683	0.688	0.949
Australian Red cows	1579	–	0.229	0.281	0.389
kg protein (variance due to SNPs 24%; posterior probability = 0.00050) ^c^					
Holstein bulls	826	0.672	0.67	0.677	0.954
Jersey bulls	221	0.716	0.711	0.724	0.949
Australian Red cows	1579	–	0.168	0.201	0.383
Fat % (variance due to SNPs 13%; posterior probability = 0.00047) ^c^					
Holstein bulls	826	0.818	0.794	0.797	0.975
Jersey bulls	221	0.631	0.649	0.681	0.973
Australian Red cows	1579	–	0.245	0.267	0.522
Protein % (variance due to SNPs 8%; posterior probability = 0.00047) ^c^					
Holstein bulls	826	0.87	0.87	0.875	0.982
Jersey bulls	221	0.793	0.819	0.835	0.979
Australian Red cows	1579	–	0.179	0.343	0.574

^a^DYD: daughter yield deviation for Holstein and Jersey bulls; YD: yield deviation for Australian Red Cows

^b^Number of validation animals

^c^The fraction of the genetic variance explained by SNPs is indicated together with the average fraction of the SNPs fitted to explain this variance (posterior prob), and the accuracy of the (D)YD used for validation

Table 3 shows the correlations between GEBV and yield-deviations when only HD 600 k SNP chip data were used in the analysis. In this case, it was possible to analyze the data by the hybrid variant of Bayes R [8], and results are shown for comparison (for reasons of computer time this analysis was performed only for milk yield). For the Bayes GC analysis, our aim was to detect the ~ 2000 SNPs with the largest effects (i.e. somewhat fewer than in the WGS data analyses), which implied that a $\pi$ value of 0.003 was used (i.e. approx. 2000/600,000). Generally, the Bayesian analyses yielded higher accuracies than the across-breed GBLUP predictions. The latter is probably because the Bayesian variable selection attempts to allocate QTL effects to SNPs that are close to the QTL, which implies that the LD between the SNPs and the QTL is more likely to persist across breeds. The latter effect is most pronounced for AR cows where all the accuracy is based on across-breed predictions, and Bayes GC and Bayes R yield 14 and 18% higher accuracy than GBLUP, respectively. For the H and J bulls, Bayes GC yielded marginally higher accuracy than the other methods, whereas for the AR cows Bayes R yielded marginally higher accuracy. Using WGS instead of HD data hardly affected prediction accuracies, although predictions for AR cows were somewhat less accurate when using HD data for four of the five traits (comparing Tables 2 and 3).

**Table 3:** Correlations between GEBV and (D)YD^a^ for milk, fat and protein yield, and fat and protein percentage using across-breed GBLUP, Bayes GC, and Bayes R predictions, when only 600 k SNP-chip data was used

	N^b^	GBLUP(AB)		Bayes GC	BayesR^c^
kg milk					
Holstein bulls	826		0.714	0.73	0.712
Jersey bulls	221		0.643	0.678	0.651
Australian Red cows	1579		0.23	0.26	0.272
kg fat					
Holstein bulls	826		0.658	0.671	
Jersey bulls	221		0.683	0.687	
Australian Red cows	1579		0.229	0.269	
kg protein					
Holstein bulls	826		0.67	0.678	
Jersey bulls	221		0.711	0.727	
Australian Red cows	1579		0.168	0.189	
Fat %					
Holstein bulls	826		0.794	0.803	
Jersey bulls	221		0.649	0.681	
Australian Red cows	1579		0.245	0.274	
Protein %					
Holstein bulls	826		0.87	0.876	
Jersey bulls	221		0.819	0.832	
Australian Red cows	1579		0.179	0.325	

^a^DYD: daughter yield deviation for Holstein and Jersey bulls; YD: yield deviation for Australian Red Cows

^b^Number of validation animals

^c^Because of the high computational costs Bayes R was only performed for milk yield

Table 4 compares the usage of computer resources by the Bayesian methods. For the HD data, Bayes GC is about four times faster than BayesR and uses eight times less memory. This difference is expected to be larger for the WGS data since Bayes GC spends less time on non-fitted SNPs, which are relatively more numerous in WGS data. In addition, the memory requirements of Bayes R would increase by a factor of ~ 8 to 9 when moving to WGS data, which is too large for current computers. When analyzing HD data, most of the memory requirements of Bayes GC are due to storing of the ${\mathbf{G}}^{-1}$ matrix and the matrix of eigenvectors ($\mathbf{E}$). The storing of the HD genotypes on 35,688 animals takes only about 5 GB of the total memory usage of 26 GB.

**Table 4:** Wall-time and random access memory (RAM) usage of the Bayesian methods when analyzing 600 k SNP-chip and WGS data

	HD SNP-chip		WGS data	
	Wall-time (h)	RAM (GB)	Wall-time (hours)	RAM (GB)
Bayes GC	36	26	133	67
Bayes R	153	201	^a^	^a^

^a^It was not possible to perform the WGS analysis

## Discussion

### Bayes GC model and computational efficiency

Genomic prediction can be described in two equivalent ways: as using SNPs to estimate the genomic relationship between the animals or as estimating the effect of SNPs that are in LD with the causal variants. If the causal variants are numerous and some have very small effects, the data may not have the power or the resolution to identify them individually. In this case, the best we can do is to estimate the effect of chromosomal segments that are present in multiple animals. In Bayes GC, this is done by the polygenic component, $u\sim N(0,\mathbf{G}{\upsigma }_{\mathrm{u}}^{2})$, which fits genomic relationships. Then, a smaller number of causal variants with larger effects are accounted for by the Bayes C component, which fits individual SNPs in high LD to important QTL. Hence, by fitting a polygenic component, fewer SNPs need to be fitted explicitly. Fitting few SNPs saves computer time since, for the vast majority of the SNPs, the solution is 0 and remains 0, i.e. updating of neither residuals nor right-hand-sides is needed.

Models that simultaneously fit a GBLUP and a BayesC term have been used before in the literature, e.g. [9, 32, 33], and have been shown to yield high prediction accuracy. Our current implementation of this model is specifically directed at the use of sequence data. To this end the algorithm for the implementation of the BayesGC model has been adjusted in several ways: (1) binary storage of the data resulted in the storing of four genotypes per byte (as in the PLINK binary format); (2) fast access to the binary genotype data stored in RAM using intrinsic Fortran90 routines and avoiding repeated centralizations of the raw genotypes; (3) using the multi-threading capacities of modern computers, several MCMC chains are run using virtually no additional computer resources, which saves computer wall-time by running many short chains in parallel instead of one long chain; and (4) using a fixed prior probability of SNPs entering the model, $\pi$, which speeds up the convergence of the chain, and thereby reduces the required length of the MCMC chain.

The following arguments justify the use of a fixed $\pi$ value: (1) a relatively small range of $\pi$ values are relevant for the Bayes GC model. If more than 5000 SNPs are needed to explain a key part of the genetic variance, the trait is so complex that a pure GBLUP model would be as accurate as Bayes GC; alternatively, if less than 1000 SNPs explain a key fraction of the variance, these could be mapped by GWAS and the mapped QTL could be explicitly accounted for in genomic predictions; (2) within this range of eligible $\pi$ values, it was expected that the actual choice of a $\pi$ value was not critical for prediction accuracies, i.e. whether one a priori expects that 2000 or 3000 out of 5 million SNPs explain an important fraction of the genetic variance will hardly affect prediction accuracies; and (3) even with a fixed $\pi$ value, the Bayes GC model can fit any distribution of SNP effects up to its fourth moment by varying the variances of the polygenic term and the fitted SNPs, assuming the distribution of SNP effects is symmetric. Zhou et al. [9] recommend BLSMM, which estimates $\pi$, ${\upsigma }_{\mathrm{s}}^{2}$, and ${\upsigma }_{\mathrm{u}}^{2}$ from the data, for its flexibility of modelling genetic effects. However, it may be questioned whether the data contain sufficient information to estimate the moments of the distribution of SNP effects beyond the fourth moment, which is also confirmed by our finding that our posterior probabilities of including a SNP into the model hardly deviated from our prior probabilities ($\pi$; see Table 2). The latter is to some degree also seen when comparing Tables 2 and 3, where the fitting of the 2500, or 2000 SNPs with the largest effect resulted in marginal differences in accuracy. If the model for the genetic effects is over-parametrized, prediction accuracies and convergence of the MCMC chain may be reduced. Bayes GC is thus very similar to BLSMM but has some features that make it especially suited for the analysis of large-scale WGS data, without sacrificing prediction accuracy. The latter makes it also suitable for the analysis of lower density genotypes.

It may seem that skipping the evaluation of the SNPs that are not in the model with probability ${v }_{i}$ slows down the movement of the MCMC chain, and thus that we need more cycles to obtain convergence. However, this is not the case for a judicious choice of ${v}_{i}$. Ideally, MH acceptance probabilities should be close to 1, which implies a move in the chain, when evaluating a SNP. Assuming that, for the less important SNPs, the posterior probability of inclusion in the model ($P{P}_{i}$) is small relative to 1, the MH acceptance probability of moving a SNP into the model is:

$${\alpha }_{{\mathrm{I}}_{i}=0\to 1}\approx \mathrm{min}(1,\frac{P{P}_{i}}{{v}_{i}}),$$

which is ~ 1 if ${v}_{i}=P{P}_{i}$. If the latter is the case, the MH-acceptance probability of moving an included SNP out of the model, ${\alpha }_{{I}_{i}=1\to 0}$, is also ~ 1. Thus, a SNP with low $P{P}_{i}$, is usually not in the model, but when it gets evaluated with probability ${v }_{i}=P{P}_{i}$, it moves into the model. After this, the SNP is evaluated in the next round again and moves out of the model. This results in, on average, $1/P{P}_{i}$ cycles where the SNP is excluded and 1 where it is included, which results in an estimate of $P{P}_{i}\approx 1/(\frac{1}{P{P}_{i}}+1)$, which is as expected. If ${v }_{i}\gg P{P}_{i}$, then ${\alpha }_{{I}_{i}=0\to 1}<1$, and the movement of the chain is not affected, but the SNP is often evaluated without moving into the model. With a very small evaluation probability ${v }_{i}\ll P{P}_{i}$, then ${\alpha }_{{I}_{i}=1\to 0}<1$, and the SNP stays often in the model once it is in it, which is to compensate for the long sequences of not being included into the model. The latter is due to the too low probability of being evaluated, ${v }_{i}$. Hence, too low ${v }_{i}$ values slow down the movement of the chain, whereas ${v }_{i}\ge P{P}_{i}$ hardly affects the expected movement of the chain. In our implementation, the smallest ${v }_{i}$ value used was 0.01, which is still 20-fold larger than the average $P{P}_{i}$ value, which equaled the prior probability $\pi =$ 0.0005 approximately (Table 2). With the lowest ranking SNPs expected to be evaluated 100 times out of 10,000 cycles, i.e. ${v}_{i}=0.01$, ~ 1 million non-fitted SNPs were evaluated per MCMC cycle, i.e. a reduction of a factor of ~ 5 compared to evaluating all SNPs. Thus, the skipping of the non-fitted SNPs with probabilities ${v}_{i}$ redirected the updating of the SNPs towards the SNPs with actual effects and sped up calculations by a factor of ~ 5. We preferred a small but non-zero probability of evaluating the lowest ranking SNPs since van den Berg et al. [11] found that dropping substantial numbers of SNPs from the analyses reduced prediction accuracy.

Memory requirements were reduced by storing four genotypes per byte of memory, following the binary storage approach of PLINK [13]. Although this increased the probability that the data could be stored in the RAM of the computer, it slowed down computations involving stored genotypes since genotypes first needed to be translated from this four genotypes per byte form into usual integers. Since a byte contained the genotypes of four animals for a particular SNP, all four genotypes were needed when evaluating this SNP (i.e. the software did not need to look-up for a particular genotype amongst those four stored in a byte since all of them were needed). These computational tunings mean that, to the best of our knowledge for the first time, a variable selection genomic prediction method could be applied to a large WGS dataset on 35,688 animals within approximately a week of computer wall time and requiring only ~ 70 Gb of RAM.

Modern high-performance computers (HPC) can run many threads in parallel and can contain large amounts of memory. However, memory intensive tasks can occupy all this memory and thereby an entire computer node, even if they do not use parallelization, i.e. most threads on the node will be idle. The availability of many threads makes the running of several (short) MCMC chains efficient. However, the memory requirements for running several single-threaded programs are high (each program stores a large matrix of genotypes and ${\mathbf{G}}^{-1}$), which will block the running of many single-threaded programs simultaneously. Bayes GC stores the genotype and ${\mathbf{G}}^{-1}$ matrices only once and then runs several parallel chains using the same stored genotypes and ${\mathbf{G}}^{-1}$. Short replicated chains can be run and their results can be combined and used for convergence diagnostics. I.e. the results across the chains are compared together with their Monte Carlo sampling error. In the current study, we used 10 parallel chains, but with modern computers many more chains can be run simultaneously, and especially for the mapping of QTL this could be advantageous (see below).

The proposal of using many, short parallel chains is limited by the burn-in cycles, i.e. each chain must be at least as long as the burn-in period. If each chain is as long as the burn-in period, the number of independent samples equals the number of chains. Parallel computations result in more CPU time per hour of wall-time, but parallel algorithms tend to require more CPU time for the same task than single-thread algorithms (due to costs of setting-up parallel tasks, waiting-time of threads, less efficient algorithms, etc.). Here, multi-threaded parallel chains contain more burn-in cycles than a single-threaded long MCMC chain. Thus, the cost of this type of parallelization is related to the number of MCMC cycles needed to obtain the next (virtually) independent MCMC sample relative to the number of the burn-in cycles. Using good starting values reduces the number of burn-in cycles, and more research on how to obtain good starting values for the MCMC chains is needed. Computations per cycle are dominated by the evaluations of the SNPs, which increase approximately linearly with the number of individuals, and less than linearly with the number of SNPs, since as the number of SNPs increases a larger fraction of the SNPs will be out of the model. Computations for the within-cycle updating of the polygenic effects increase quadratically with the number of individuals. The eigen-decomposition of the ${{\mathbf{R}}^{1/2}\mathbf{G}}^{-1}{\mathbf{R}}^{1/2}$ matrix, which is of size number of animals, is with current algorithms limited to ~ 100,000 animals, but computation costs are small relative to those of the MCMC computations. Storage of the eigen-vectors and the ${\mathbf{G}}^{-1}$ matrix increases quadratically with the number of animals, and storage of the genotypes increases with the product of the number of animals times the number of SNPs. Generally, computation costs are high, but feasible for large numbers of individuals (< 100,000) with (imputed) WGS data.

### Bayes GC to map QTL

The Bayes GC model resembles the standard GWAS model, since GWAS models generally fit a polygenic component and a single SNP effect simultaneously. Hence, the Bayes GC model may be seen as an extension of the standard GWAS model towards fitting many SNPs simultaneously. This increases mapping precision, since a QTL effect will not yield mapping signals across long distances, because a closer SNP, which is in stronger LD with the QTL, will be fitted and pick-up the QTL’s effect. The latter is not the case for the typical GWAS methods that fit the SNPs one-by-one. However, the complex inheritance pattern at the beginning of BTA14, which suggests the presence of several causal mutations (e.g. [24]), seemed too complicated to unravel by simply running Bayes GC. Fitting the K232A mutation in *DGAT1* [25] as a fixed effect in the Bayes GC model might help to locate the other QTL, but this was beyond the scope of the current study.

Our approach to fine-scale mapping was to first identify (250-kb) regions with a large variance of local GEBV. This gives clearer QTL signals than a genome-wide search for high posterior probability SNPs for two reasons. First, if there are multiple SNPs in high LD with the causal variant, no one SNP may have a high posterior probability. Second, a SNP may have its posterior probability overestimated, for instance, because the MCMC chain has not converged due to the SNP being stuck in the model for too many MCMC cycles. The latter is remedied substantially by running multiple chains. We constructed a 95% credibility interval surrounding the SNP with the highest posterior probability, by identifying within every MCMC sample the SNP that was closest to this position estimate.

The construction of 95% posterior probability intervals is often performed by summing the posterior probabilities of individual SNPs in the region until they exceed 0.95 (e.g. [34]). However, when a SNP that explains a large QTL moves to a new position, first a second SNP is fitted in the region, and next the first SNP, after a number of MCMC cycles of competition between the SNPs, is sampled out of the model. Hence, during many cycles there are two or more SNPs fitted to explain the QTL, which increases posterior probabilities, and makes the posterior probability intervals unrealistically short, i.e. the estimates of the intervals are anti-conservative. In fact, using this approach one could fit intervals that contain more than 100% posterior probability, which is not possible under the assumption of only one QTL in the region (but the GC model may fit more QTL). Our 95% credibility interval based on the nearest fitted SNP contained a total sum of posterior probabilities of the SNPs contained in the interval of on average 1.50 for the intervals fitted in this study. Hence, our alternative way of fitting posterior probability intervals is more conservative.

Additional file 4: Tables S1 and S2 show examples where a single SNP reaches a posterior probability higher than 0.95 and both ways of estimating confidence intervals would result in an interval containing only a single SNP. Such a single SNP credibility interval indicates that the implicated SNP clearly has the strongest association with a QTL (or several QTL in the region). However, since the causative polymorphism may not be in our data, it may differ from the implicated SNP and thus reside outside the single SNP credibility interval. Hence, the 95% credibility interval holds strictly for the SNPs associated with the causative mutation, and not for the causative mutation itself which may not be in our data and outside this interval. If, however, the 95% posterior probability contains many SNPs in a LD-block that also contains the causative mutation, it becomes unlikely that the causative mutation is among the most peripheric variants on this LD-block. Hence, if the 95% credibility interval contains many SNPs (ideally > 100, which may be the case for sequence data), this interval contains the causative mutation with a probability of ~ 95%. Therefore, if the 95% credibility interval contains many SNPs (> 100 SNPs), it may be interpreted as containing the causative mutation with a probability of ~ 95%, otherwise it contains with a 95% probability the SNP that is most associated with the QTL. The 95% credibility intervals for the fat percentage QTL in Fig. 3 and [see Additional file 3: Figures S9 to S12] contained on average 398 SNPs (ranging from 36 to 750) and contained the corresponding COJO SNPs [24], and the *DGAT1* and *GHR* mutations.

The Manhattan plots in Fig. 1, and the detailed mapping results of the six largest QTL for fat percentage on BTA2, 5, 11, 14, and 20 (Figs. 2, 3, 4) and [see Additional file 1: Figures S1 to S4, Additional file 2: Figures S5 to S8, and Additional file 3: Figures S9 to S12], demonstrated the mapping precision of Bayes GC. These mapping results and the top 10 of the 250-kb regions with the largest variances of local GEBV, aligned closely with the QTL mapping results from the meta-analysis of van den Berg et al. [24]. This may be partly due to the fact that the current Holstein and Jersey data also participated in this meta-analysis, but the current dataset is still considerably smaller than that of [24] and their data may have contained information on QTL that were not present in our study. Hence, considering the size of the current data, and that only two breeds were used for QTL mapping (the Australian Red data were masked), the two studies agree remarkably well. In addition, the mapping precision achieved by combining imputed WGS data across breeds and MCMC-based variable selection methods that fit the most associated SNPs (as shown in Figs. 3 and 4) and [see Additional file 3: Figures S9 to S12 and Additional file 4: Tables S1 and S2] seemed remarkably high. When applied to milk yield, Bayes GC was able to map three QTL within a 5-Mb region on BTA20. This included the F27Y mutation in the *GHR* gene [31], a QTL at 30.145126 Mb, which was also found in [24], and a new QTL affecting milk production at position 34.501126 Mb on BTA20.

### Genomic prediction

For the Jersey and Australian Red data, the average increases in accuracy were (across the traits) 2.5 and 16.5%, respectively, when applying Bayes GC instead of GBLUP (ignoring the increase for protein percentage, which was exceptionally large). In the case of Holsteins, which have a large reference population, this average increase in accuracy was only 1.2%. The relative large increase in accuracy of AR cows was probably because the GBLUP accuracy was low at an average of 0.218 (excluding protein percentage), i.e. there was a lot of room for improvement. The genomic prediction accuracies in Table 2 may be considered relative to the accuracies of the (D)YD. Since the (D)YD own accuracy reflects the maximum accuracy by which the (D)YD can be predicted by a (perfect) GEBV. The accuracies of DYD are all higher than 0.949, so that scaling by the accuracies of DYD hardly makes any difference to the results. For AR cattle, scaling by the accuracies of the YD results in most of the accuracies of Bayes GC GEBV lying within a narrow range from 0.51 to 0.6 (across the traits), i.e. across-breed predictions of AR cows in the absence of a within breed reference population were moderately accurate.

The Jersey and, particularly, the Holstein bulls already had high prediction accuracies (0.6 to 0.8), and data/model improvements only resulted in moderate increases of their GEBV accuracies. Comparing Tables 2 and 3, it seems that it was also possible to achieve very similar improvements in accuracies by Bayesian variable selection methods using HD data, although for AR cows the accuracy was about 2% higher when using WGS data. Similar increases in accuracy when including sequence data were found by Zhang et al. [35]. Possible explanations for why the major increase in genotype density from HD to WGS results only in minor improvements in prediction accuracy are: (1) WGS genotypes are substantially less accurate than HD SNP-chip genotypes (due to imputation errors and sequencing errors); (2) the HD SNP-chip is sufficiently dense to detect SNPs in high across-breed-LD with the QTL and that WGS data result in a surplus of such high LD SNPs (the variable selection problem becomes more challenging whereas the improvements in LD are only moderate); and (3) only a limited fraction of the variance is explained by QTL with large effects that persist across breeds. For AR cows, the accuracy of across-breed GBLUP was low and Bayesian variable selection methods in combination with WGS data resulted in larger increases in prediction accuracies than for J and H animals which have rather large within-breed reference populations.

In spite of the implemented speed improvements in the Bayes GC software, it is too slow for practical evaluations of breeding values. When using the GBLUP approach for genetic evaluations, one could give extra weight to the SNPs according to their posterior probability of being included into the model when setting up the $\mathbf{G}$-matrix. This requires the availability of WGS data on all training animals and selection candidates, which may be obtained by genotype imputation using HD-SNP chip data (and probably lower density SNP chip data). The computational costs of WGS genotype imputation and data storage for all animals in the genetic evaluations will be high. An alternative approach is to use the QTL mapping results from the WGS analyses, possibly augmented with results from other analyses, and to add top-SNPs from each QTL region to the SNP-chip [36]. This is expected to extract most of the information from the WGS data for genomic predictions, and accuracies of prediction may be close to those obtained here, especially when substantial numbers of top-SNPs can be identified (~ 2000 to 3000).

## Conclusions

Across-breed variable selection based genomic prediction improved prediction accuracies relative to GBLUP, especially in the absence of a sizeable within-breed reference population. Using WGS instead of 600 k SNP-chip data yielded on average a 3% accuracy improvement for Australian Red cows. The combination of across-breed WGS data and a variable selection genomic prediction method proved remarkably effective for the fine-mapping of QTL.

## Supplementary Information

**Additional file 1: Figure S1.** Manhattan plots of the variance of the local GEBV within 250-kb regions for kg milk. **Figure S2.** Manhattan plots of the variance of the local GEBV within 250-kb regions for kg fat. **Figure S3.** Manhattan plots of the variance of the local GEBV within 250-kb regions for kg protein. **Figure S4.** Manhattan plots of the variance of the local GEBV within 250-kb regions for protein percentage.

**Additional file 2: Figure S5.** Manhattan plot of the variance of the local GEBV within 250-kb regions for fat percentage on BTA2. **Figure S6.** Manhattan plot of the variance of the local GEBV within 250-kb regions for fat percentage on BTA5. **Figure S7.** Manhattan plot of the variance of the local GEBV within 250-kb regions for fat percentage on BTA11. **Figure S8.** Manhattan plot of the variance of the local GEBV within 250-kb regions for fat percentage on BTA14.

**Additional file 3: Figure S9.** Fine scale map of the posterior probabilities of the SNPs that affect fat percentage in the neighborhood of the fat percentage QTL on BTA2 shown in Figure S1.1. The blue bar denotes the 95% credibility interval for the QTL, and the red dot the position of the COJO SNP detected by [24]. **Figure S10.** Fine scale map of the posterior probabilities of the SNPs for affecting fat percentage in the neighborhood of the fat percentage QTL on BTA5 shown in Fig. 2. The blue bar denotes the 95% credibility interval for the QTL, and the red dot the position of the COJO SNP detected by [24]. **Figure S11.** Fine scale map of the posterior probabilities of the SNPs for affecting fat percentage in the neighborhood of the fat percentage QTL on BTA11 shown in Figure S12. The blue bar denotes the 95% credibility interval for the QTL, and the red dot the position of the COJO SNP detected by [24]. **Figure S12.** Fine scale map of the posterior probabilities of the SNPs for affecting fat percentage in the neighborhood of DGAT1 on BTA14 shown in Figure S1.3. The blue bar denotes the 95% credibility interval for the QTL, and the red dots denote the positions of two COJO SNPs detected by [24].

**Additional file 4: Table S1.** Positions (Mb) of QTL for fat yield and their 95% credibility interval in the top-10 of 250-kb regions explaining most variance of local EBV (excluding regions neighboring the main QTL on BTA14). **Table S2.** Positions of QTL (Mb) for protein yield and their 95% credibility interval in the top-9 of 250-kb regions explaining most variance of local EBV (excluding the regions neighboring the main QTL on BTA14).
