# Supplementary material for: On the use of whole-genome sequence data for across-breed genomic prediction and fine-scale mapping of QTL
Source: Genet Sel Evol. 2021 Feb 26;53:19. doi: 10.1186/s12711-021-00607-4 (PMC7908738; doi:10.1186/s12711-021-00607-4)

Supplementary Material 1

Figure S1.1. Manhattan plots of the variance of the local GEBV within 250 kb regions for kg milk.

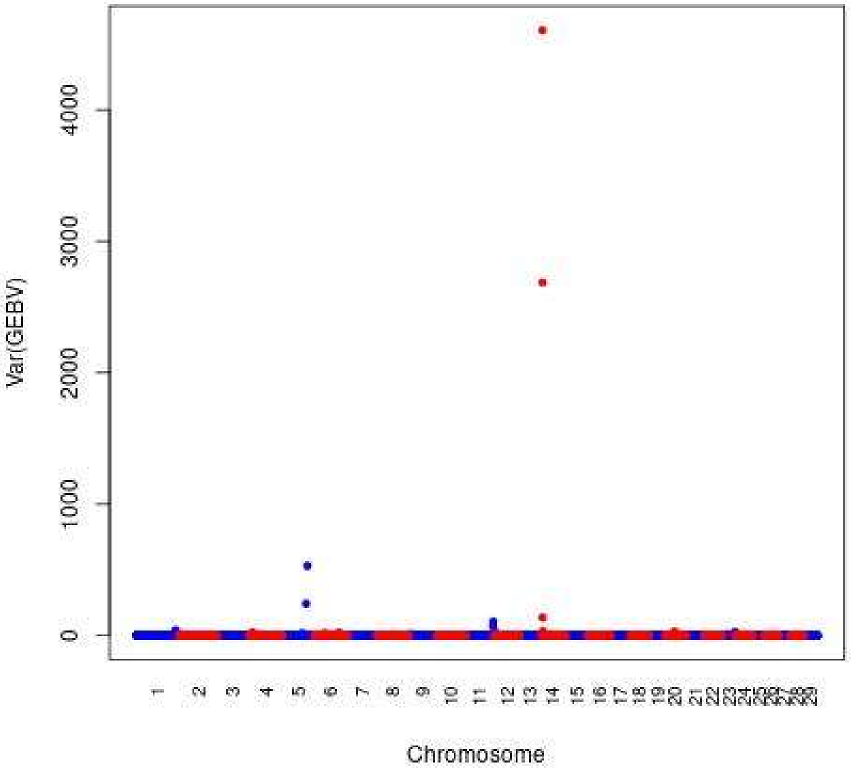

Figure S1.2. Manhattan plots of the variance of the local GEBV within 250 kb regions for kg fat.

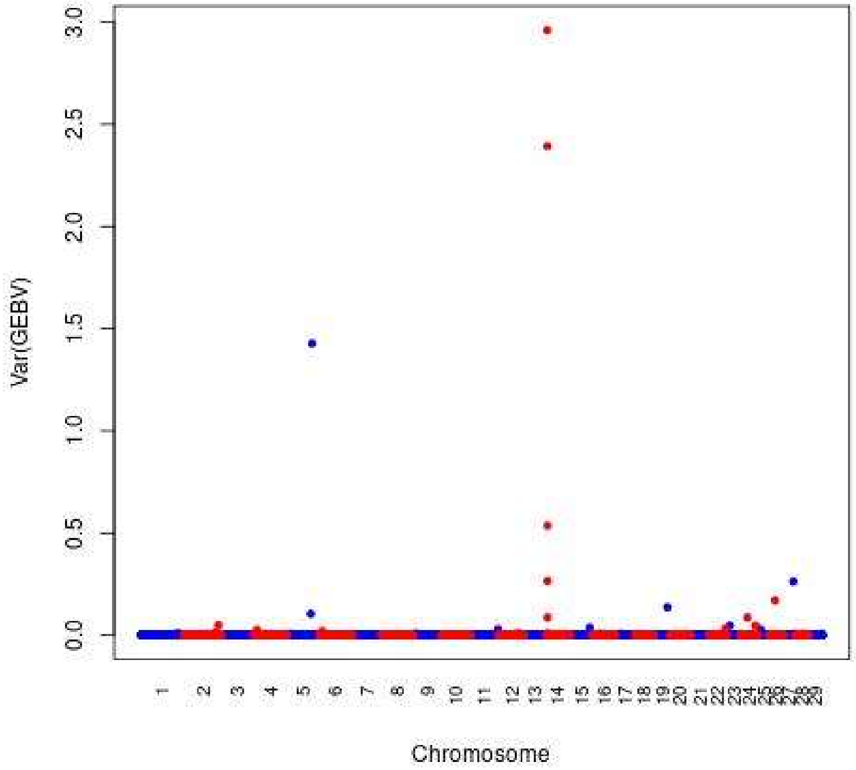

Figure S1.3. Manhattan plots of the variance of the local GEBV within 250 kb regions for kg protein.

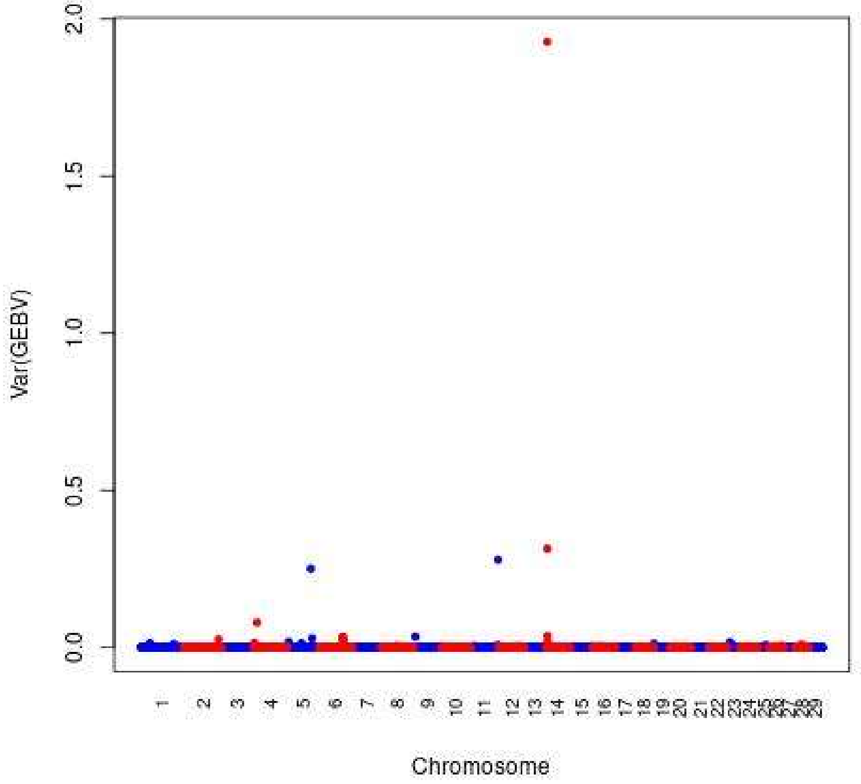

Figure S1.4. Manhattan plots of the variance of the local GEBV within 250 kb regions for protein percentage.

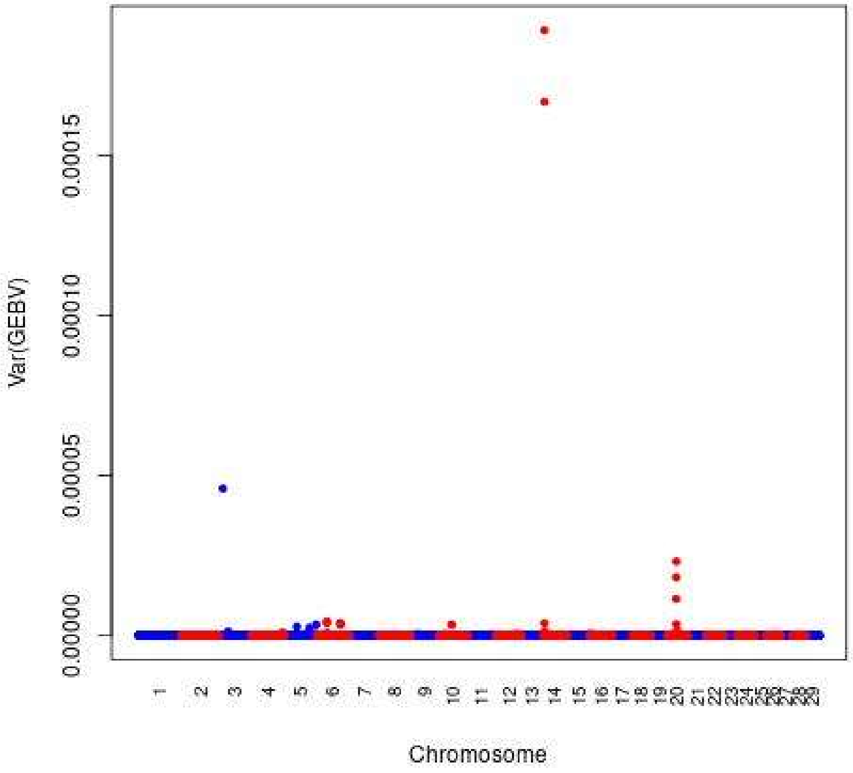

Supplement: Supplementary file 1 — Additional file 1: Figure S1. Manhattan plots of the variance of the local GEBV within 250-kb regions for kg milk. Figure S2. Manhattan plots of the variance of the local GEBV within 250-kb regions for kg fat. Figure S3. Manhattan plots of the variance of the local GEBV within 250-kb regions for kg protein. Figure S4. Manhattan plots of the variance of the local GEBV within 250-kb regions for protein percentage. [file 12711_2021_607_MOESM1_ESM.pdf]
