# Supplementary material for: On the use of whole-genome sequence data for across-breed genomic prediction and fine-scale mapping of QTL
Source: Genet Sel Evol. 2021 Feb 26;53:19. doi: 10.1186/s12711-021-00607-4 (PMC7908738; doi:10.1186/s12711-021-00607-4)

Supplementary material 2

Figure S2.1: Manhattan plot of the variance of the local GEBV within 250 kb regions for fat percentage on BTA 2.

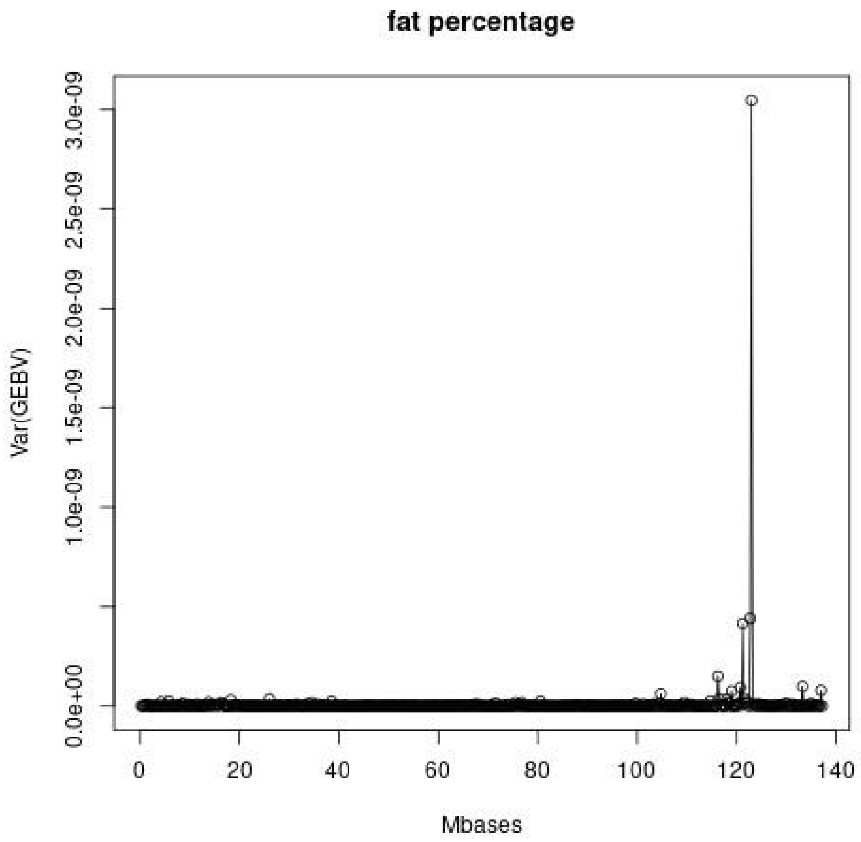

Figure S2.2: Manhattan plot of the variance of the local GEBV within 250 kb regions for fat percentage on BTA 5.

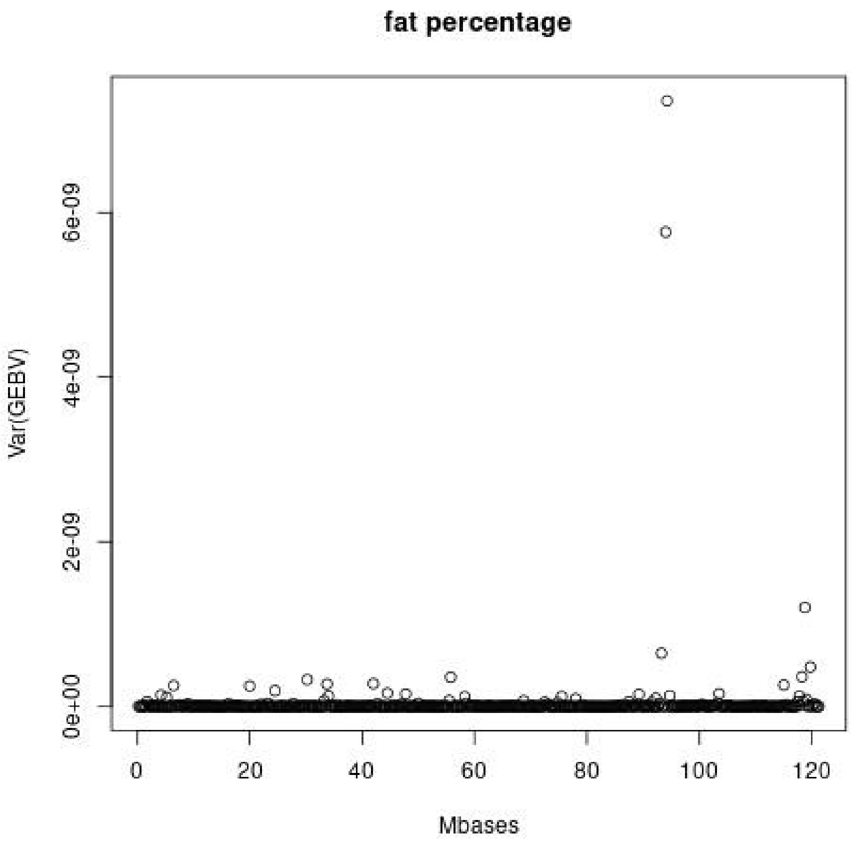

Figure S2.3. Manhattan plot of the variance of the local GEBV within 250 kb regions for fat percentage on BTA 11.

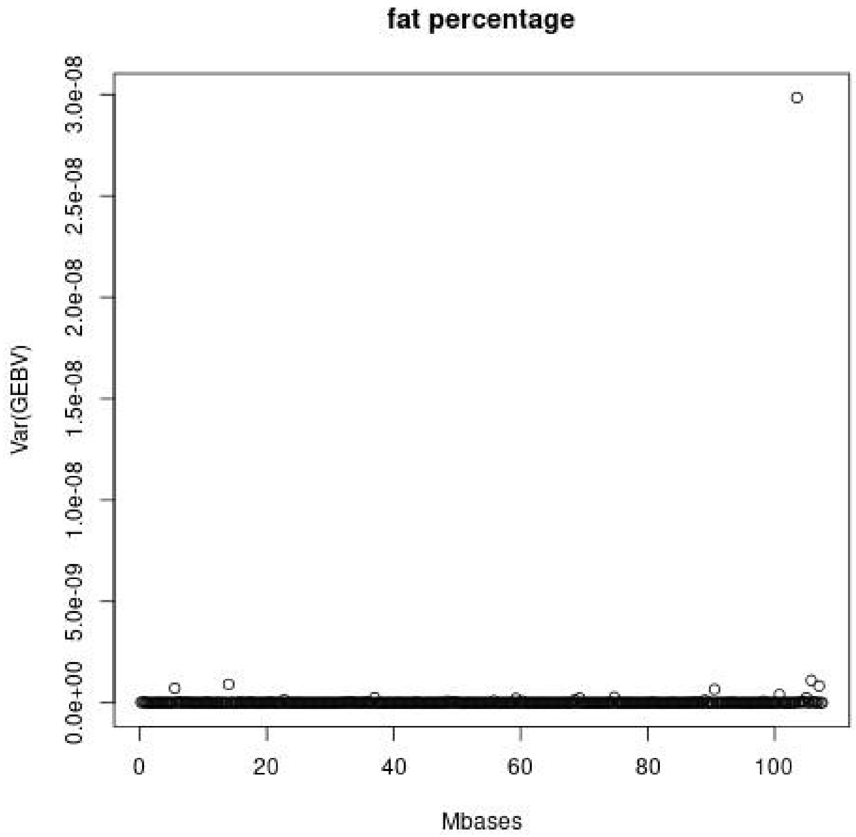

Figure S2.4: Manhattan plot of the variance of the local GEBV within 250 kb regions for fat percentage on BTA 14.

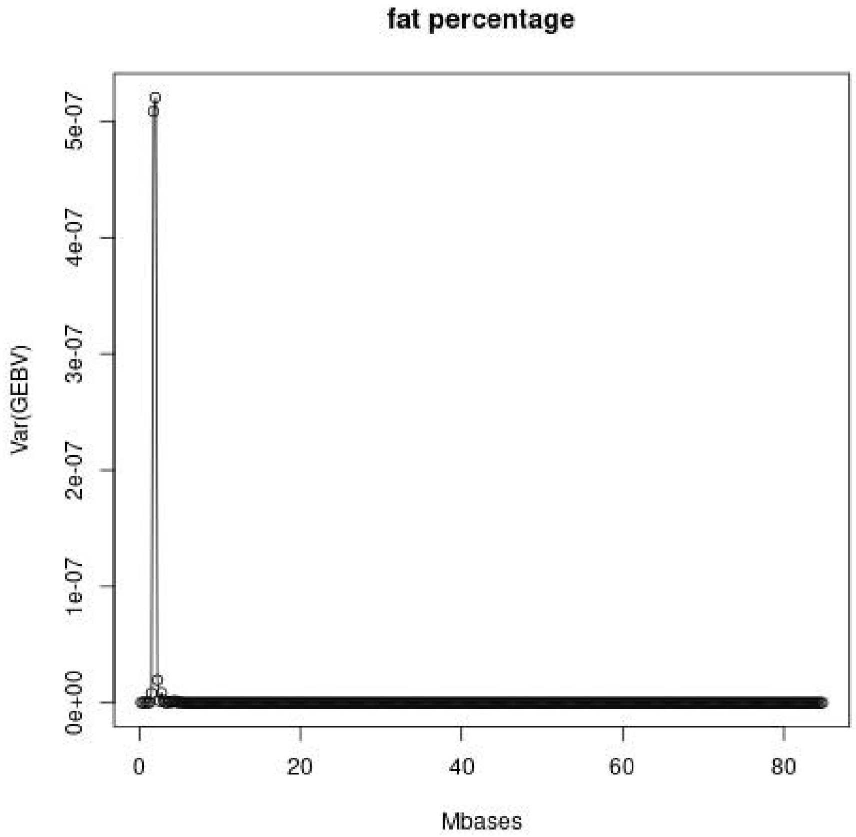

Supplement: Supplementary file 2 — Additional file 2: Figure S5. Manhattan plot of the variance of the local GEBV within 250-kb regions for fat percentage on BTA2. Figure S6. Manhattan plot of the variance of the local GEBV within 250-kb regions for fat percentage on BTA5. Figure S7. Manhattan plot of the variance of the local GEBV within 250-kb regions for fat percentage on BTA11. Figure S8. Manhattan plot of the variance of the local GEBV within 250-kb regions for fat percentage on BTA14. [file 12711_2021_607_MOESM2_ESM.pdf]
