# Supplementary material for: On the use of whole-genome sequence data for across-breed genomic prediction and fine-scale mapping of QTL
Source: Genet Sel Evol. 2021 Feb 26;53:19. doi: 10.1186/s12711-021-00607-4 (PMC7908738; doi:10.1186/s12711-021-00607-4)

Supplementary Material 3

Figure S3.1. Fine scale map of the posterior probabilities of the SNPs for affecting fat percentage in the neighborhood of the fat percentage QTL on BTA 2 depicted in Figure S1.1. The blue bar denotes the 95% credibility interval for the QTL, and the red dot the position of the COJO SNP detected by [24].

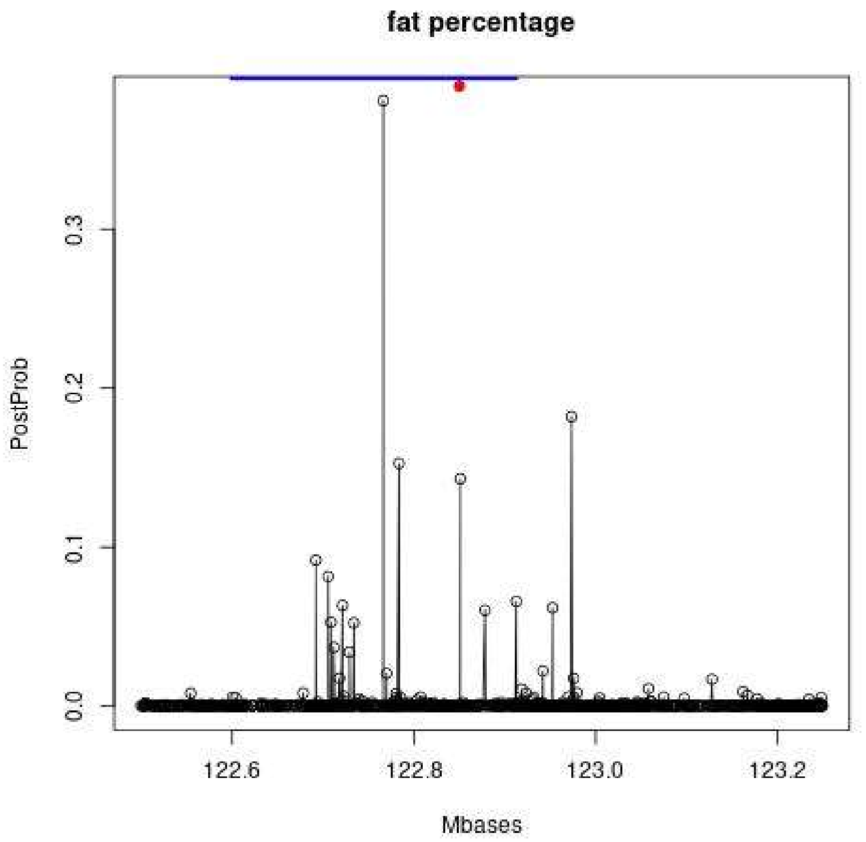

Figure S3.2. Fine scale map of the posterior probabilities of the SNPs for affecting fat percentage in the neighborhood of the fat percentage QTL on BTA 5 depicted in Figure 2. The blue bar denotes the 95% credibility interval for the QTL, and the red dot the position of the COJO SNP detected by [24].

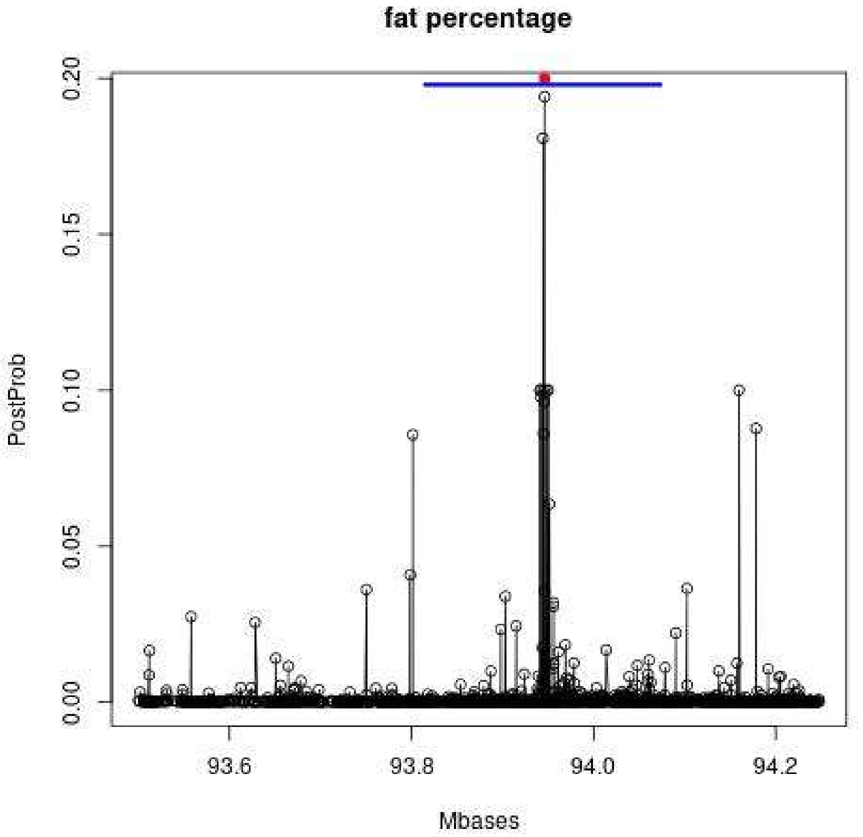

Figure S3.3. Fine scale map of the posterior probabilities of the SNPs for affecting fat percentage in the neighborhood of the fat percentage QTL on BTA 11 depicted in Figure S1.2. The blue bar denotes the 95% credibility interval for the QTL, and the red dot the position of the COJO SNP detected by [24].

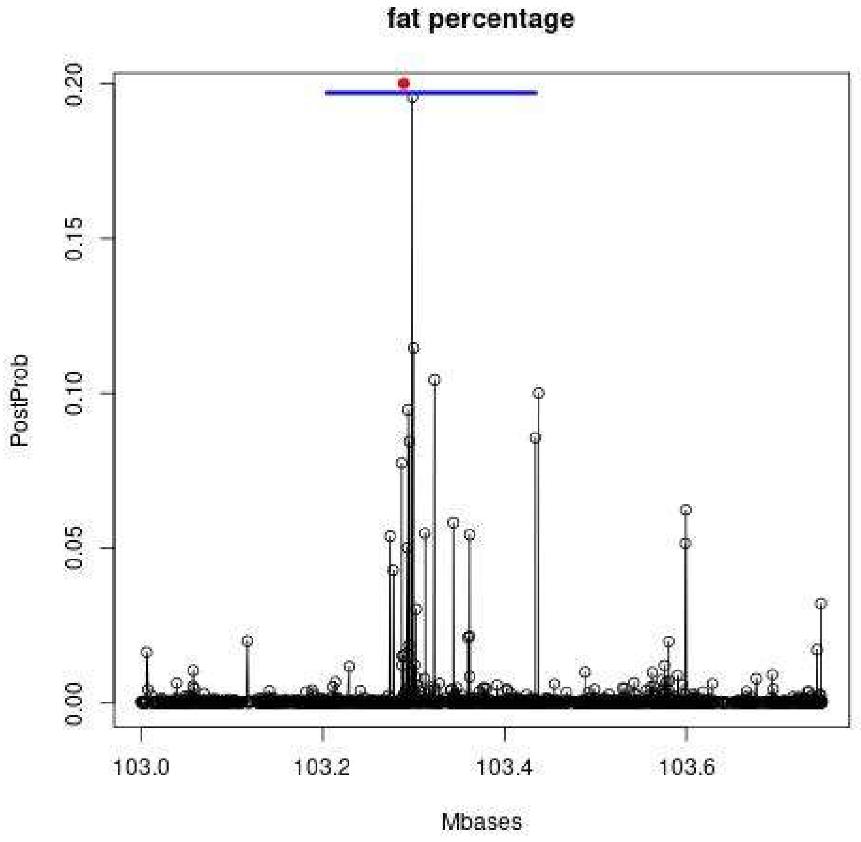

Figure S3.4. Fine scale map of the posterior probabilities of the SNPs for affecting fat percentage in the neighborhood of DGAT1 on BTA 14 depicted in Figure S1.3. The blue bar denotes the 95% credibility interval for the QTL, and the red dots the positions of two COJO SNPs detected by [24].

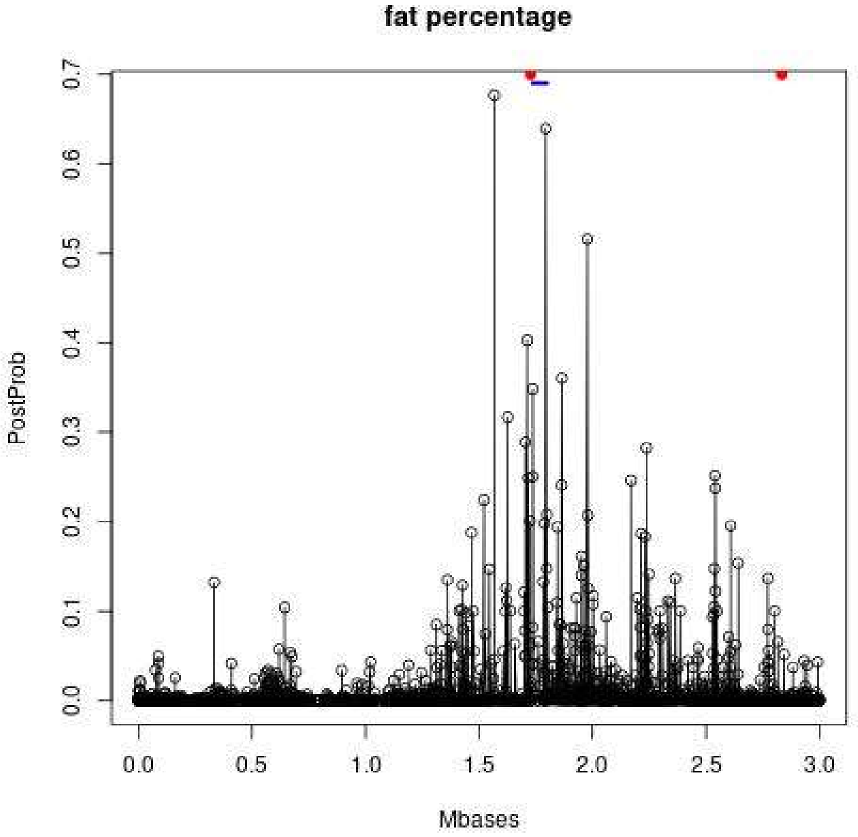

Supplement: Supplementary file 3 — Additional file 3: Figure S9. Fine scale map of the posterior probabilities of the SNPs that affect fat percentage in the neighborhood of the fat percentage QTL on BTA2 shown in Figure S1.1. The blue bar denotes the 95% credibility interval for the QTL, and the red dot the position of the COJO SNP detected by [24]. Figure S10. Fine scale map of the posterior probabilities of the SNPs for affecting fat percentage in the neighborhood of the fat percentage QTL on BTA5 shown in Fig. 2. The blue bar denotes the 95% credibility interval for the QTL, and the red dot the position of the COJO SNP detected by [24]. Figure S11. Fine scale map of the posterior probabilities of the SNPs for affecting fat percentage in the neighborhood of the fat percentage QTL on BTA11 shown in Figure S12. The blue bar denotes the 95% credibility interval for the QTL, and the red dot the position of the COJO SNP detected by [24]. Figure S12. Fine scale map of the posterior probabilities of the SNPs for affecting fat percentage in the neighborhood of DGAT1 on BTA14 shown in Figure S1.3. The blue bar denotes the 95% credibility interval for the QTL, and the red dots denote the positions of two COJO SNPs detected by [24]. [file 12711_2021_607_MOESM3_ESM.pdf]
