# Supplementary material for: On the use of whole-genome sequence data for across-breed genomic prediction and fine-scale mapping of QTL
Source: Genet Sel Evol. 2021 Feb 26;53:19. doi: 10.1186/s12711-021-00607-4 (PMC7908738; doi:10.1186/s12711-021-00607-4)
Supplement: Supplementary file 4 — Additional file 4: Table S1. Positions (Mb) of QTL for fat yield and their 95% credibility interval in the top-10 of 250-kb regions explaining most variance of local EBV (excluding regions neighboring the main QTL on BTA14). Table S2. Positions of QTL (Mb) for protein yield and their 95% credibility interval in the top-9 of 250-kb regions explaining most variance of local EBV (excluding the regions neighboring the main QTL on BTA14). [file 12711_2021_607_MOESM4_ESM.pdf]

Supplementary Material 4

Table S4.1: Positions (Mb) of QTL for fat yield and their 95% credibility interval in the top-10 of 250kb regions explaining most variance of local EBV (excl. regions neighboring the main QTL at BTA14).[1]

Chromosome	Top SNP Position [2]	95%-credibility interval
14	1.793616	1.793616 - 1.793616
5	93.948357	93.948357 - 93.948718
27	36.155097	36.131079 – 36.179449
26	21.138509	21.111181 – 21.184734
19	51.319673	51.319673 - 51.319673
24	24.772882	24.755903 – 24.779981
14	2.770991	2.715689 – 2.801735
5	88.776125	88.623921 – 88.959862
23	12.066876	11.900889 – 12.276830
24	54.487057	54.434317 – 54.572910

[1]SNPs in regions with highest variance of local EBV are ranked first.

[2] SNP with highest posterior probability of being included in the model.

Table S4.2: Positions of QTL (Mb) for protein yield and their 95% credibility interval in the top-9 of 250kb regions explaining most variance of local EBV (excl. regions neighboring the main QTL at BTA14).[1]

Chromosome	Top SNP Position[2]	95%- credibility interval
14	1.716713	1.712619 - 1.736599
11	103.301757	103.299604 – 103.317601
5	88.823164	88.823164 – 88.830128
4	10.140203	10.057523 – 10.228753
9	8.744481	8.673870 – 8.816700
6	87.188371	87.101329 – 87.347102
6	83.596196	83.402189 – 83.878307
14	2.933370	2.775557 – 3.136689
5	93.815566	93.564084 – 93.948718

[1]SNPs in regions with highest variance of local EBV are ranked first.

[2] SNP with highest posterior probability of being included in the model.
